# Comparison of Tissue Factors in the Ontogenetic Aspects of Human Cholesteatoma

**DOI:** 10.3390/diagnostics14060662

**Published:** 2024-03-21

**Authors:** Kristaps Dambergs, Gunta Sumeraga, Māra Pilmane

**Affiliations:** 1 Department of Otorhinolaryngology, Riga Stradiņš University, Pilsonu Street 13, LV-1002 Riga, Latvia; gunta.sumeraga@rsu.lv; 2 Children’s Clinical University Hospital, Vienības Gatve 45, LV-1004 Riga, Latvia; 3 Department of Morphology, Institute of Anatomy and Anthropology, Riga Stradiņš University, LV-1007 Riga, Latvia; mara.pilmane@rsu.lv

**Keywords:** cholesteatoma, defensins, cytokines, Ki-67, transcription factors, vascular endothelial growth factor, Sonic hedgehog, metalloproteases, adults, children

## Abstract

Background: An acquired cholesteatoma is a benign but locally aggressive lesion in the middle ear. It is characterized by chronic inflammation and the destruction of surrounding bone. Therefore, the aim of this study was to compare defensins HβD-2 and HβD-4; pro- and anti-inflammatory cytokines IL-1α and IL-10; proliferation marker Ki-67; transcription factor NF-κβ; angiogenetic factor VEGF; Sonic hedgehog gene protein SHH; and remodeling factors MMP-2, MMP-9, TIMP-2, and TIMP-4 in adult and pediatric cholesteatoma tissue, and to compare these groups with control skin tissue. Methods: The study included 25 cholesteatoma tissue material samples from children, 25 from adults, and 7 deep external ear canal skin samples from cadavers. The tissues were stained immunohistochemically and evaluated using semi-quantitative methods. Nonparametric tests, such as the Kruskal–Wallis test and Spearman rank correlation, were used. Results: There were no statistically discernible differences between the adult and children groups when comparing the relative numbers of factor-positive cells. Conclusions: There are no histopathological differences between adult and children cholesteatoma tissues.

## 1. Introduction

An acquired cholesteatoma is a benign but potentially destructive lesion of the middle ear that is composed of a hyperproliferating keratinized squamous epithelium [1]. A common symptom is conductive or mixed hearing loss, purulent discharge from the ear, and ear pain in rare cases. There are several serious and life-threatening complications associated with this disease, including meningitis, brain abscess, facial nerve paresis, and sigmoid sinus thrombosis [2]. Children are affected by cholesteatoma at a rate of 3 to 15 per 100,000 children, and adults are affected at a rate of 9 per 100,000 people, with the overall incidence being 7 per 100,000 people in Europe [3,4]. Due to the above-mentioned characteristics, we evaluated the tissue factors responsible for inflammation, rapid growth, and destruction, which can be associated with the pathogenesis of cholesteatomas.

One of the main features of immunity is a response to invading pathogens such as bacteria, and cholesteatoma is characterized by chronic bacterial infection. Human beta defensin 2 (HβD-2) is known to be a local antibacterial peptide that was first found in human skin [5], but it can be secreted by various types of epithelial cells in a human organism [6]. Studies have revealed that HβD-2 can be activated by IL-1α and *P. aeruginosa*; furthermore, NF-κβ from activated B lymphocytes binds to HβD-2 and induces it [7]. Unlike HβD-1, which is always expressed, HβD-2 is expressed in epithelial cells when stimulated by pro-inflammatory cytokines [8]. Previous studies have shown up-regulation of HβD-2 in human cholesteatomas compared to skin [9,10]. Additionally, HβD-2 is very effective in killing *P. aeruginosa* (up to 90% of colony formation units), which is also found in chronic ear infections [5]. In a similar manner to HβD-2, human beta defensin 4 (HβD-4) is active against *P. aeruginosa*, but less effective against *S. pneumoniae* and *S. aureus* [11]. However, there are limited data on its role in human cholesteatoma. Our previous studies suggest that HβD-4 is less expressed in human cholesteatoma than HβD-2 [12,13].

Cholesteatoma presents with chronic inflammation and often episodes of exacerbation [14]. The potent pro-inflammatory cytokine is interleukin 1 alfa (IL-1α); this is expressed in various types of cells—macrophages, lymphocytes, neutrophils, fibroblasts, epithelial and endothelial cells, keratinocytes, and other cell types [15]. In cholesteatoma, IL-1α is associated with the destruction of the surrounding middle ear bone and cholesteatoma growth [16,17]. Unlike IL-1α, interleukin 10 (IL-10) is a potent anti-inflammatory cytokine that is essential to prevent inflammatory pathologies, and it is one of the tissue factors that helps maintain and/or restore homeostasis in the human organism [18,19]. In addition to immune effector cells such as T-helpers, monocytes, macrophages, mast cells, granulocytes, and others, epithelial cells and keratinocytes are also capable of producing IL-10 [20]. The disturbance between pro-inflammatory and anti-inflammatory cytokines may be one of the reasons for its aggressiveness [21]. 

Cholesteatoma proliferates uncontrollably, which is one of the key factors for its many complications [22]. The most used marker to detect proliferation in cholesteatoma cells is Ki-67 [23]. This is a protein found in the cell nucleus and is present in the active phases but absent in the resting phase of the cell cycle [24]. In addition to indicating proliferation activity, Ki-67 has been associated with the degree of bone resorption in cholesteatoma [25]. 

Nuclear factor kappa beta (NF-κβ) was first identified in B lymphocytes as a transcription factor that stimulates the κ light chain of immunoglobulin [26]. NF-κβ is almost always present in all processes in the human organism. It regulates hundreds of genes, mostly related to immune and inflammatory processes. In addition to this, NF-κβ regulates the gene responsible for cell proliferation and apoptosis [27]. In human cholesteatoma, NF-κβ is associated with cell proliferation and inflammatory processes [14,28]. 

Angiogenesis has been associated with the expansion of cholesteatomas [29], and vascular endothelial growth factor (VEGF) is one of the most potent angiogenetic factors [30]. VEGF is produced by many different cell types, including keratinocytes, macrophages, tumor cells, platelets, and endotheliocytes [30]. VEGF has previously been shown to be one of the tissue factors that regulates angiogenesis in human cholesteatomas [29]. 

To examine cholesteatomas from an ontogenetic point of view, we chose to evaluate the Sonic hedgehog (SHH) gene protein. SHH is associated with the development of structures in the external, middle, and inner ear [31,32,33]. The disturbance of SHH gene signaling can cause external and middle ear pathologies [34]. However, there is scarce information about the role of SHH in cholesteatoma development. Our previous studies showed up-regulation of the SHH gene protein in pediatric and adult cholesteatoma cases compared with control skin [12,13].

Most of the complications of cholesteatoma in the middle ear arise from bone erosion [35]. Remodeling factors matrix metalloproteinase 2 and 9 (MMP-2 and MMP-9) can cause bone destruction in the case of cholesteatoma [36,37]. MMP-2 and MMP-9 belong to the gelatinase family [38]. MMP-2 is a gelatinase A, and can cleave gelatin (a denatured collagen), collagens (type I, IV, V), vitronectin, and elastin [39]. MMP-2 activates and inhibits inflammation by releasing pro-inflammatory cytokines, such as IL-1α, and regulates angiogenesis [40]. A similar role for MMP-2 is observed in cholesteatoma tissue [36]. MMP-9 is a gelatinase B that cleaves collagen types IV, V, VII, X, XIV, laminin, and fibronectin [39,41]. MMP-9 releases the active form of VEGF, and is directly associated with angiogenesis [42]. The tissue inhibitors of metalloproteinases 2 and 4 (TIMP-2, TIMP-4) inhibit MMP-2 and MMP-9, respectively [43]. Previous studies have shown that an imbalance between MMP and TIMP can lead to tissue degradation in the structures of the middle ear in patients with cholesteatoma [44,45]. 

This is one of the most extensive studies in terms of the number of tissue factors used to compare cholesteatoma in ontogenesis. The chosen combination of tissue factors helps to evaluate the pathogenesis of cholesteatoma from several perspectives—defense against pathogens, regulation and presentation of inflammation, proliferation, angiogenesis, tissue remodulation, and presentation of gene proteins in cholesteatoma tissue. Comparing many tissue markers in one study and in the same collected cholesteatoma material can provide more precise conclusions about the disease’s pathogenesis than comparing several but different studies; this is because the methods and tissues will always have differences. We believe that using a variety of tissue factors and comparing both cholesteatoma groups are the strongest aspects of our study. Additionally, to our knowledge, several tissue factors such as SHH, HβD-4, and TIMP-4 have not been analyzed in cholesteatoma tissue.

As a result of the small amount of studies comparing cholesteatoma in children and adults, especially at the histological level, there are uncertainties between the authors about whether pediatric cholesteatoma differs from adult cholesteatoma (in recidivism rate or aggressiveness), and if any differences exist at the cellular level. Therefore, the main purpose of this study was to compare defensins HβD-2 and HβD-4; pro- and anti-inflammatory cytokines IL-1α and IL-10; proliferation marker Ki-67; transcription factor NF-κβ; angiogenetic factor VEGF; Sonic hedgehog gene protein SHH; and remodeling factors MMP-2, MMP-9, TIMP-2, and TIMP-4 in adult and pediatric cholesteatoma tissue, and to compare these groups with control skin tissue.

## 2. Materials and Methods

### 2.1. Tissue Samples

This study was carried out between November 2019 and June 2023. It was approved by the ethics committee of Riga Stradiņš University (5 September 2019; no. 6-2/7/4) and implemented in accordance with the Declaration of Helsinki. All of the patients and/or their legal caregivers provided informed consent to participate in the study. The purpose of the study was fully explained to the patients. 

The cholesteatoma tissue was gathered during surgery at the Children’s Clinical University Hospital in Riga and at P. Stradiņš Clinical University Hospital, by the same doctor in each hospital. Immunohistochemical staining of the tissue was carried out at the Department of Morphology of Riga Stradiņš University, Latvia.

The children group consisted of 25 patients—15 boys and 10 girls (ages varied from 5 to 17 years, mean age 12.72 years). In the adult group, 25 patients were included in the study, 11 male and 14 female (ages ranged from 19 to 75 years, mean age 39.44 years). The inclusion criterion was acquired cholesteatoma. Eight children and six adults were excluded from this study. The exclusion reasons were incomplete cholesteatoma material after staining with hematoxylin and eosin (cholesteatoma without matrix and/or perimatrix), which was not valid for further analysis. The cholesteatomas in both patient groups were proven clinically and histologically. 

The control group consisted of cadaver external ear’s meatal skin material. We collected the tissue material during autopsies from 10 different cadavers (ages ranging from 35 to 70 years). The cadavers were part of the collection of the Institute of Anatomy and Anthropology. The autopsies were performed within 12 h after biological death. The ethics committee of Riga Stradiņš University approved the use of the bodies of the deceased (29 October 2022; 2-PĒK-4/475/2022). The inclusion criteria were the following: no chronic ear disease; no chronic skin disease; no blunt-force trauma to the external ear region. Three skin samples presented with insufficient epidermal or dermal layers for further analysis and were thus excluded.

### 2.2. Immunohistochemical Analysis

The tissues were fixed in a mixture of 2% formaldehyde and 0.2% picric acid in 0.1 M phosphate buffer (pH 7.2). Afterwards, they were rinsed in Tyrode buffer (content: NaCl, KCl, CaCl_2__2H_2_O, MgCl_2__6H_2_O, NaHCO_3_, NaH_2_PO_4__H_2_O, glucose) containing 10% saccharose for 12 h, and then embedded in paraffin.

Thin, 3 µm sections were cut. To remove paraffin, xylene (BC-5L, Biognost, Zagreb, Croatia) was used; to dehydrate tissue sections, alcohol 96° was applied. The HiDef Detection™ HRP Polymer System (954D-30, Cell Marque, Rocklin, CA, USA) was used to ready the slides for IHC staining, and to identify the following markers in the tissue samples: HβD-2 (sc-20798, rabbit, working dilution 1:200, Santa Cruz Biotechnology, Inc., Dallas, TX, USA); HβD-4 (sc-59496, mouse, working dilution 1:50, Santa Cruz Biotechnology, Inc., Dallas, TX, USA); IL-1α (sc-9983, mouse, working dilution 1:50, Santa Cruz Biotechnology, Inc., Paso Robles, CA, USA); IL-10 (ab34843, rabbit, working dilution 1:400, Abcam, Cambridge, UK); Ki-67 (1325506A, rabbit, 1:100, Cell Marque, Rocklin, CA, USA); NF-κβ (sc-109; rabbit, 1:200 dilution, Santa Cruz Biotechnology, Inc., Paso Robles, CA, USA); vascular endothelial growth factor (VEGF) (orb191500, rabbit, 1:100, Biorbyt Ltd., Cambridge, UK); SHH (LS-C49806, rabbit, 1:100, LifeSpan BioSciences, Inc., Seattle, WA, USA); MMP2 (sc-53630, mouse, 1:100, Santa Cruz Biotechnology, Inc., Dallas, TX, USA); MMP-9 (sc-10737, rabbit, 1:100, Santa Cruz Biotechnology, Inc., Santa Cruz, Dallas, TX, USA); TIMP2 (sc-21735, mouse, 1:100, Santa Cruz Biotechnology, Inc., Dallas, TX, USA); and TIMP-4 (sc-30076, rabbit, 1:100, Santa Cruz Biotechnology, Inc., Santa Cruz, Dallas, TX, USA).

Furthermore, tissue samples were rinsed in wash buffer (TRIS; T0083, Diapath S.p.A., Martinengo, Italy) two times for 5 min, then placed in a microwave oven for 20 min in boiling EDTA buffer (T0103, Diapath S.p.A., Martinengo, Italy) and cooled to 65 °C for approximately 20 min. Then, the material was placed in TRIS wash buffer and blocked with 3% peroxidase block (925B-02, Cell Marque, Rocklin, CA, USA) for 10 min. All of the antibodies used in this study were diluted with antibody diluent (938B-05, Cell Marque, Rocklin, CA, USA).

For antibodies of rabbit or mouse origin, the HiDef Detection^TM^ HRP polymer system (954D-30, Cell Marque, Rocklin, CA, USA) was used. The slides were rinsed five times (5 min each) with TRIS buffer solution. Then, HiDef Detection^TM^ reaction amplifier (954D-31, Cell Marque, Rocklin, CA, USA) was applied for 10 min at room temperature. Afterwards, the material was rinsed five times (5 min each time) in distilled water. After that, HRP chromogen (together with DAB buffer) (957D-30, Cell Marque, Rocklin, CA, USA) was used for 3–5 min. Chromogen was made fresh for each application. The preparations were washed 5 times with TRIS buffer. The slides were placed in a slide basket and immersed in filtered hematoxylin for 30–60 s. After staining with hematoxylin, the slides were rinsed five times in distilled water and dehydrated in alcohol (95% and 100% for 3 min), and then immersed in three containers with xylene (5 min each) and, finally, dried and covered with Pertex^®^ (00801-EX, HistoLab, Västra Frölunda, Gothenburg, Sweden) glue. The positive controls were developed in accordance with company guidelines and negative controls—excluding primary antibodies (Supplementary Materials, Figure S1). 

Two independent morphologists used a semi-quantitative method [46] (Table 1) to analyze the slides using light microscopy. Both morphologists evaluated the slides simultaneously and, after the evaluation, agreed on the results. There were no significant differences between both evaluations. The evaluation of each patient’s micrograph consisted of grading the appearance of positively stained cells in the visual field. Multiple visual fields were evaluated in each micrograph. Structures in the visual field were labeled as seen in Table 1. The micrographs of each criterion as an example are found in the Supplementary Materials (Figure S2).

A Leica DC 300F digital camera and image processing software Image-Pro Plus, Version 10 (Media Cybernetics, Inc., Rockville, MD, USA) were used for microphotograph development.

### 2.3. Statistical Analysis

Data processing was carried out using SPSS software version 29.0 (IBM, Chicago, IL, USA). To adapt the semi-quantitative method for the SPSS software, the results were labeled as follows: no positive structures (0) in the visual field were labeled “0” (0 = 0); 0-0/+ = 0.25, 0/+ = 0.5; 0/+-+ = 0.75; + = 1; +/++ = 1.5; ++ = 2; ++/+++ = 2.5; +++ = 3; +++/++++ = 3.5; and ++++ = 4. Nonparametric tests were used for the ordinal data. Spearman’s rank correlation was used to detect correlations within each group. The Kruskal–Wallis test was used to detect differences between all groups (children, adults, control).

The Spearman rank correlation coefficient was used to determine the correlations between factors and were valued as follows: r = 0–0.2, a very weak correlation; r = 0.2–0.4, a weak correlation; r = 0.4–0.6, a moderate correlation; r = 0.6–0.8, a strong correlation; and r = 0.8–1.0, a very strong correlation. The significance level of the test was selected at 5% (*p*-value < 0.05).

## 3. Results

The results section is divided into subsections. In each subsection, the results of analyzed tissue factors are described. The same results are summarized in Table 2 and Table S1 (Supplementary Materials). Furthermore, the microphotographs are presented as an example of each cell marker. At the end of the results section, statistically drawn results are summarized in Table 3, Table 4 and Table 5. 

### 3.1. Description of the Analyzed Tissue

The cholesteatoma tissue samples that were included in this study all contained three layers. The most inner part, the cystic layer, is composed of an anucleated keratin mass; the middle part, the matrix, is formed from the epithelial cells and has the same layers as the unchanged skin but which are hyperproliferated; the outer part is the perimatrix, composed of loose connective tissue, collagen fibers, fibrocytes, and different inflammatory cells such as lymphocytes, neutrophils, and plasma cells. 

**Figure 1:**
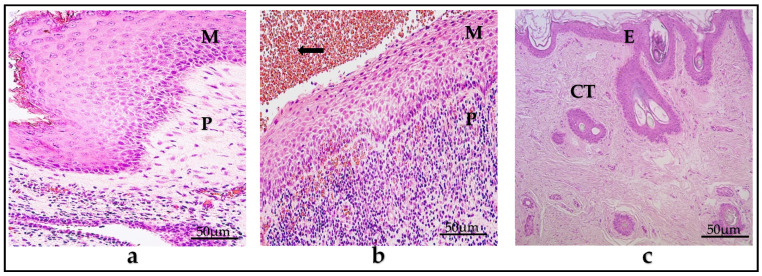
(**a**–**c**) Histological micrographs. (**a**) Child cholesteatoma. (**b**) Adult cholesteatoma. Note the following in both (**a**,**b**) micrographs: M—matrix, which is hyperproliferated squamous epithelium. P—perimatrix, consisting mostly of inflammatory cells. Black arrow—erythrocytes. (**c**) Unchanged skin, E—epithelium, CT—subepithelial connective tissue. Hematoxylin and eosin. ×200.

The control group comprised deep, intact external ear canal skin, consisting of an unchanged stratified squamous epithelial layer and non-inflammatory subepithelial connective tissue (Figure 1a–c).

### 3.2. Immunohistochemistry of Defensins

The mean number of HβD-2-positive cells in the pediatric cholesteatoma matrix was few to moderate (+/++) and had a range from zero (0) positive cells to moderate to numerous (++/+++) in the visual field. In the perimatrix, the mean number was few (+), within a range from no (0) positive cells to moderate (++) in the visual field.

The average values of HβD-4 in the children’s cholesteatomas’ matrix and perimatrix were occasional to few (0/+), with these ranging from zero (0) to numerous (+++) positive structures in the visual field. 

In the adult group, the mean numbers of HβD-2-positive cells were few to moderate (+/++) in the matrix and few (+) in the perimatrix, ranging from an occasional amount (0/+) to moderate to numerous (++/+++) in the matrix, and from zero (0) to moderate (++) in the perimatrix. On average, the HβD-4-positive cells in the matrix were few (+), but in the perimatrix were occasional (0/+) and these varied from zero (0) to moderate (++) HβD-4-positive cells in the visual field.

**Figure 2:**
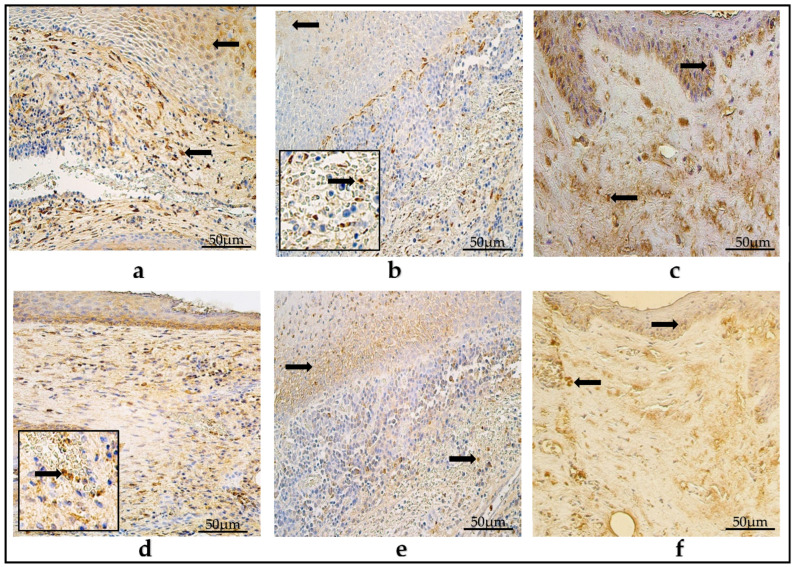
(**a**–**f**) Immunohistochemical micrographs. (**a**) Child cholesteatoma. Moderate number of HβD-2-positive cells (arrows) in the matrix and perimatrix. (**b**) Adult cholesteatoma. Few to a moderate number of HβD-2-positive cells (arrow) in the matrix and perimatrix (magnification + arrow). (**c**) Skin. Few HβD-2-positive cells (arrows) in the epithelium and occasional in the connective tissue. HβD-2 IHC, X200. (**d**) Child cholesteatoma. A moderate number of HβD-4-positive cells in the matrix and a few-to-moderate number (magnification + arrow) in the perimatrix. (**e**) Adult cholesteatoma. A moderate-to-numerous number of HβD-4-positive cells in the matrix and few in the perimatrix (arrow). (**f**) Skin. Few HβD-4-positive cells (arrows) in the epithelium and few in the connective tissue. HβD-4 IHC, ×200.

In the control group, in the epithelium, there were few (+) HβD-2-positive cells on average, but in connective tissue, there was a range of zero to few (0-0/+) positive cells. The values varied from zero (0) to moderate (++) positive epitheliocytes in the epithelium, and from zero (0) to few (0/+) positive cells in the connective tissue. The mean number of HβD-4-containing cells in the epithelium was few (+) and varied within a range from few (+) to moderate (++); however, in the connective tissue, there were an occasional amount (0/+), varying from zero (0) to few (+) positive cells (Table 2) (Figure 2a–f). 

Complete relative values of each factor are shown in Supplementary File S1 (Table S1).

**Table 2:** Median values of immunohistochemical evaluation.

Groups	HβD-2		HβD-4		IL-1α		IL-10		Ki-67		NF-κβ		VEGF		SHH		MMP-2		MMP-9		TIMP-2		TIMP-4	
	M	P	M	P	M	P	M	P	M	P	M	P	M	P	M	P	M	P	M	P	M	P	M	P
Children	+/++	+	0/+-+	0/+-+	+/++	+	+/++	+	0/+	0/+-+	++	+	++	0/+-+	++	+/++	+/++	+	0/+-+	0/+	+	0/+	++/+++	++
Adults	+/++	+	+	0/+	+/++	+	+/++	+	0/+	0/+	++	+	+/++	0/+-+	++/+++	+/++	+/++	+	0/+	0/+	+	0/+	++/+++	++
Control	E	CT	E	CT	E	CT	E	CT	E	CT	E	CT	E	CT	E	CT	E	CT	E	CT	E	CT	E	CT
	+	0-0/+	+	0/+	0/+-+	0/+-+	+/++	+/++	0-0/+	0-0/+	0/+	0/+-+	++/+++	0/+-+	+/++	0/+-+	+	+	+	0/+-+	+/++	0/+-+	++	+/++

Abbreviations: M—matrix; P—perimatrix; E—epithelium; CT—connective tissue; HβD-2—human beta defensin 2; HβD-4—human beta defensin 4; IL-1α—interleukin 1; IL-10—interleukin 10; Ki-67—proliferation marker; NF-κβ—nuclear factor kappa beta; VEGF—vascular endothelial growth factor; Shh—Sonic hedgehog gene protein; MMP-2—matrix metalloproteinase 2; MMP-9—matrix metalloproteinase 9; TIMP-2—tissue inhibitor of metalloproteinase-2; TIMP-4—tissue inhibitor of metalloproteinase-4; 0 = no positive structures, 0-0/+ = no to occasional positive structures, 0/+ = occasional positive structures, 0/+-+ = occasional-to-few positive structures, + = few positive structures, +/++ = few-to-moderate number of positive structures, ++ = moderate number of positive structures, ++/+++ = moderate-to-numerous positive structures, +++ = numerous positive cells, +++/++++ = numerous-to-abundant structures, ++++ = abundance of positive structures in the visual field.

### 3.3. Immunohistochemistry of Cytokines

In the children group, the mean counts of IL-1α- and IL-10-positive cells in the matrix were few to moderate (+/++), and in the perimatrix, there were few (+), with these varying from zero (0) to numerous (+++). 

In the adult group, the average values of IL-1α- and IL-10-containing cells were few to moderate (+/++) and few (+) in the matrix and perimetrix, respectively. These ranged from zero (0) to numerous (+++) factor-positive cells in the visual field. 

**Figure 3:**
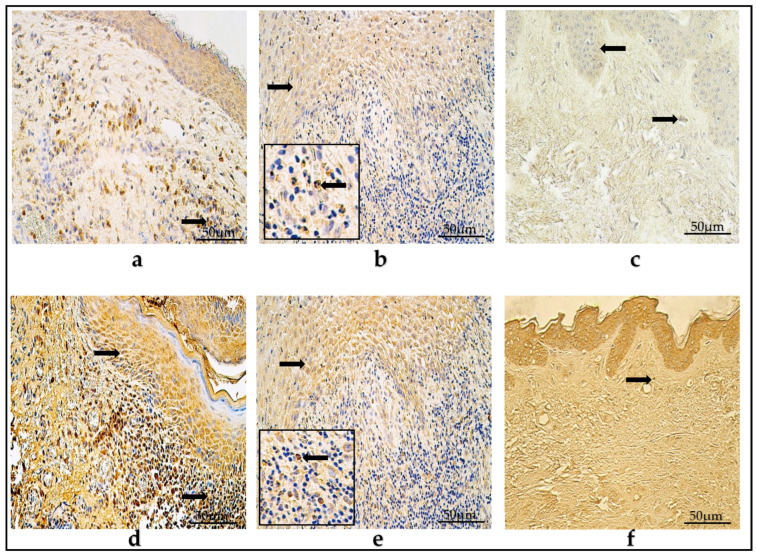
(**a**–**f**) Immunohistochemical micrographs. (**a**) Child cholesteatoma. A moderate-to-numerous number of IL-1α -positive cells in the matrix and a few-to-moderate number in the perimatrix (arrow). (**b**) Adult cholesteatoma. A moderate-to-numerous number of IL-1α -positive cells in matrix (arrow) and an occasional amount in the perimatrix (magnification + arrow). (**c**) Skin. Few IL-1α -positive cells (arrows) in the epithelium and few in the connective tissue. IL-1α IHC, X200. (**d**) Child cholesteatoma. A numerous amount of IL-10-positive cells (arrows) in the matrix and a moderate-to-numerous amount in the perimatrix. (**e**) Adult cholesteatoma. A moderate-to-numerous number of IL-10-positive cells in the matrix (arrow) and few in the perimatrix (magnification + arrow). (**f**) Skin. Numerous IL-10-positive cells in the epithelium and few (arrow) in the connective tissue. IL-10 IHC, ×200.

In the control group, the IL-1α-positive cells in the epithelium and connective tissue were occasional to few (0/+-+), and, on average, the IL-10-positive cells were few to moderate (+/++) in the epithelium and connective tissue, displaying a variance from occasional (0/+) to moderate to numerous (++/+++) factor-containing cells (Table 2) (Figure 3a–f).

### 3.4. Ki-67 Immunohistochemistry

In the children group, the mean amount Ki-67-positive cells was occasional (0/+) in the matrix, while practically no cells were detected (0-0/+) in the perimatrix. The values ranged from zero (0) to moderate (++) positive cells amongst the group.

In the adult group, the mean number of Ki-67-containing cells was occasional (0/+), with the range varying from zero (0) to few to moderate (+/++).

**Figure 4:**
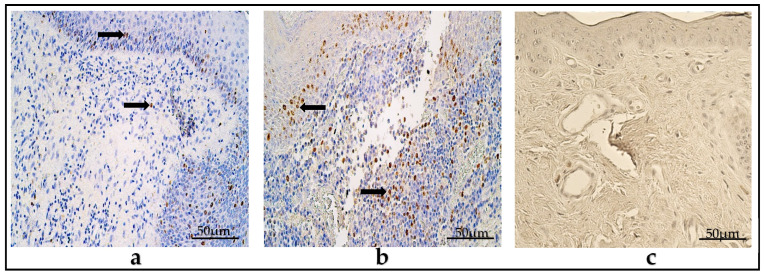
(**a**–**c**) Immunohistochemical micrographs. (**a**) Child cholesteatoma. Few Ki-67-positive cells (arrows) in the matrix and an occasional number in the perimatrix. (**b**) Adult cholesteatoma. A few-to-moderate amount of Ki-67-positive cells (arrows) in the matrix and the perimatrix. (**c**) Skin. No Ki-67-positive cells were detected in epithelium and connective tissue. Ki-67 IHC, ×200.

In the control group, Ki-67-positive cells averaged from zero to an occasional number of factor-containing cells (0-0/+) in the epithelium and connective tissue (Table 2) (Figure 4a–c). 

### 3.5. NF-κβ Immunohistochemistry

In the children group, the average number of NF-κβ-positive cells in the matrix was moderate (++), while in the perimatrix there were few (+), within a range that varied from zero (0) to numerous (+++) factor-positive cells. 

In the adult group, the average number of NF-κβ-containing cells in the matrix was moderate (++), while in the perimatrix there were few (+), within a range that varied from zero (0) to numerous (+++) NF-κβ-positive cells.

**Figure 5:**
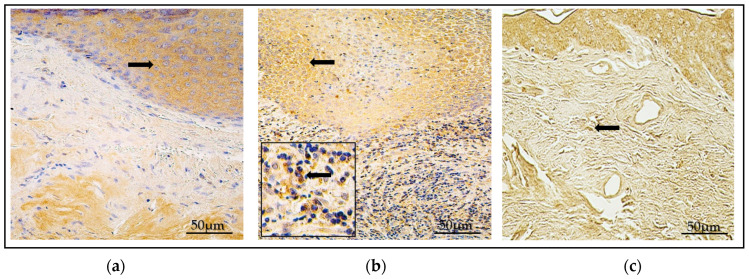
(**a**–**c**) Immunohistochemical micrographs. (**a**) Child cholesteatoma. A moderate-to-numerous amount of NF-κβ -positive cells (arrows) in the matrix and zero in the perimatrix. (**b**) Adult cholesteatoma. A numerous amount of NF-κβ-positive cells in the matrix (arrow) and the perimatrix (magnification + arrow). (**c**) Skin. A moderate-to-numerous amount of NF-κβ -positive cells in the epithelium and few (arrow) in the connective tissue. NF-κβ IHC, ×200.

In the control group, the mean number of NF-κβ-positive cells in the epithelium was occasional to few (0/+-+), while in the connective tissue there was an occasional amount (0/+), within a range that presented a variance from zero (0) to moderate (++) NF-κβ-containing cells (Table 2) (Figure 5a–c). 

### 3.6. VEGF Immunohistochemistry

The mean number of VEGF-containing cells in the children group was moderate (++) in the matrix, with an occasional amount to few (0/+-+) in the perimatrix, within a range that varied from zero (0) to a numerous amount and also to an abundance (+++/++++) of VEGF-positive cells.

In the adult group, the average values were few to moderate (+/++) in the matrix and occasional to few (0/+-+) in the perimatrix, within a range that presented a variance of between zero (0) factor-positive cells to numerous (+++).

**Figure 6:**
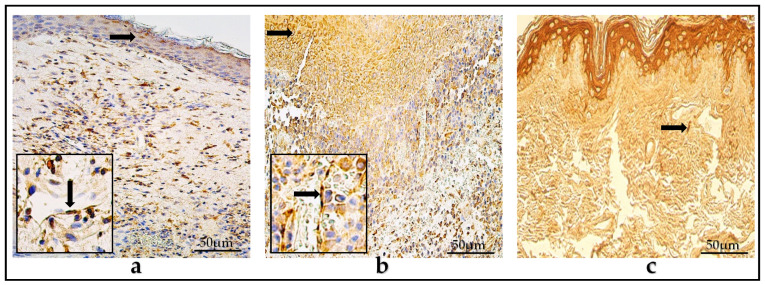
(**a**–**c**) Immunohistochemical micrographs. (**a**) Child cholesteatoma. A few-to-moderate number of VEGF-positive cells in the matrix (arrow) and a moderate amount in the perimatrix (magnification + arrow—positive endotheliocyte). The arrow in the perimatrix points out positive endotheliocyte. (**b**) Adult cholesteatoma. Numerous-to-abundant amount of VEGF-positive cells in matrix (arrow) and a moderate amount in the perimatrix (magnification + arrow—positive endotheliocyte). (**c**) Skin. Numerous VEGF-positive cells in the epithelium and few (arrow) in the connective tissue. VEGF IHC, ×200.

On average, the control group showed a moderate-to-numerous (++/+++) number of VEGF-containing cells in the epithelium, with an occasional amount to few (0/+-+) in the connective tissue. The range was from zero (0) to numerous (+++) factor-positive cells in the visual field (Table 2) (Figure 6a–c).

### 3.7. SHH Immunohistochemistry

In the children group, the number of SHH-positive cells in the matrix, on average, was moderate (++), while in the perimatrix, there was few to a moderate number (+/++). The amount varied within a range of zero (0) factor-positive cells to numerous to abundant (+++/++++) in the visual field.

The adult group showed a moderate-to-numerous (++/+++) amount of SHH-containing cells in the matrix and a few-to-moderate (+/++) amount in the perimatrix, within a range from an occasional number (0/+) to an abundance (++++).

**Figure 7:**
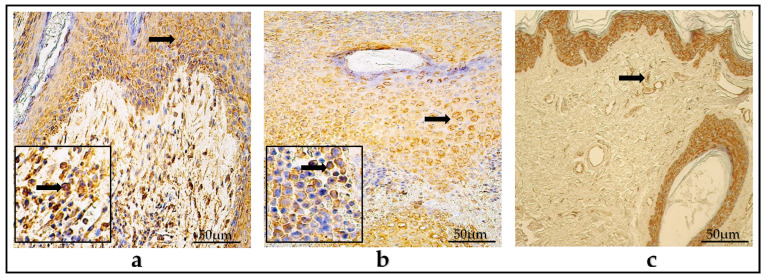
(**a**–**c**) Immunohistochemical micrographs. (**a**) Child cholesteatoma. Numerous SHH-positive cells in the matrix (arrow) and in the perimatrix (magnification + arrow). (**b**) Adult cholesteatoma. Numerous SHH-positive cells in matrix (arrow) and few in the perimatrix (magnification + arrow). (**c**) Skin. Numerous to abundant SHH-positive cells in the epithelium and an occasional number (arrow) in the connective tissue. SHH IHC, ×200.

In the control group, on average, SHH-positive cells were few to moderate (+/++) in the epithelium, with occasional to few (0/+-+) in the connective tissue. The amount of factor-positive cells presented a range of variance from zero (0) to numerous to abundant (+++/++++) (Table 2) (Figure 7a–c).

### 3.8. Immunohistochemistry of Tissue-Remodeling Factors

In the children group, the mean number of MMP-2-containing cells was few to moderate (+/++) in the cholesteatoma’s matrix, and few (+) in the perimatrix. The number of factor-positive cells varied from zero (0) to numerous (+++).

**Figure 8:**
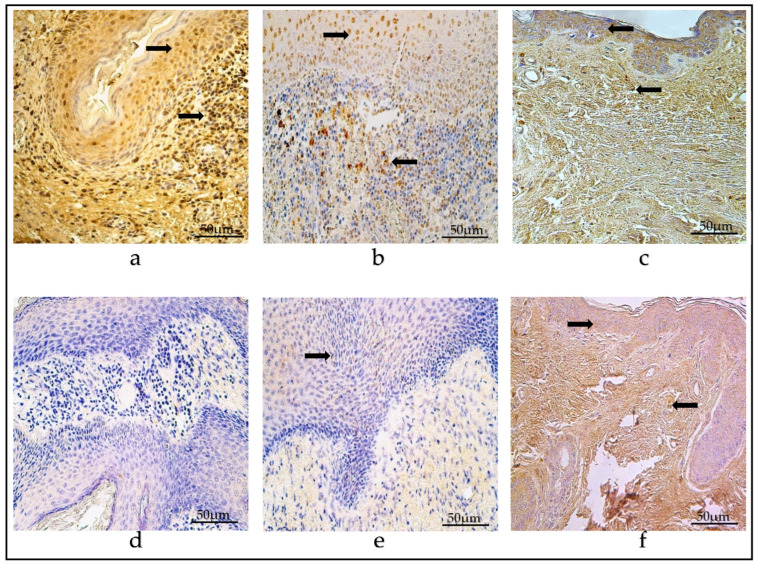
(**a**–**f**). Immunohistochemical micrographs. (**a**) Child cholesteatoma. Moderate to numerous number of MMP-2-positive cells (arrows) in the matrix and in the perimatrix. (**b**) Adult cholesteatoma. Moderate number of MMP-2-positive cells (arrows) in the matrix and few to moderate in the perimatrix. (**c**) Skin. Moderate to numerous MMP-2-positive cells (arrows) in the epithelium and few in the connective tissue. MMP-2 IHC, X200. (**d**) Child cholesteatoma. A lack of MMP-9-positive cells in the matrix and in the perimatrix. (**e**) Adult cholesteatoma. Few MMP-9-positive cells (arrow) in the matrix and their absence in the perimatrix. (**f**) Skin. Moderate number of MMP-9-positive cells (arrows) in the epithelium and in the connective tissue. MMP-9 IHC, ×200.

The average numbers of MMP-9-positive cells were occasional to few (0/+-+) and an occasional amount (0/+) in the matrix and perimatrix, respectively, within a range from zero (0) to moderate (++) MMP-9-containing cells (Figure 8a,d).

**Figure 9:**
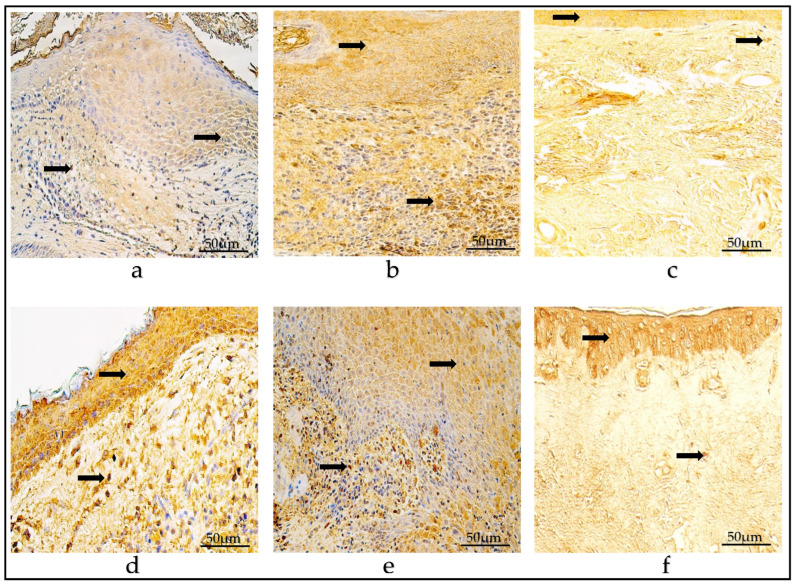
(**a**–**f**) Immunohistochemical micrographs. (**a**) Child cholesteatoma. Moderate number of TIMP-2-positive cells (arrows) in the matrix and few in the perimatrix. (**b**) Adult cholesteatoma. Numerous to abundant number of TIMP-2-positive cells (arrows) in the matrix and moderate in the perimatrix. (**c**) Skin. Numerous TIMP-2-positive cells (arrows) in the epithelium and few in the connective tissue. TIMP-2 IHC, X200. (**d**) Child cholesteatoma. Numerous-to-abundant TIMP-4-positive cells (arrows) in the matrix and moderate-to-numerous in the perimatrix. (**e**) Adult cholesteatoma. Moderate-to-numerous TIMP-4-positive cells (arrow) in matrix and in the perimatrix. (**f**) Skin. Moderate-to-numerous TIMP-4-positive cells (arrows) in the epithelium and few in the connective tissue. TIMP-4 IHC, ×200.

TIMP-2 averaged few (+) and an occasional amount (0/+) of factor-positive cells in the matrix and perimatrix, respectively. The mean value of TIMP-4-positive cells in the matrix was moderate to numerous (++/+++), and was moderate (++) in the perimatrix. The range of numbers of TIMP-2- and TIMP-4-positive cells varied from zero (0) to numerous to abundant (+++/++++) amongst the group subjects (Figure 9a,d). 

In the adult group, the mean number of MMP-2-positive cells in the cholesteatoma’s matrix was few to moderate (+/++), and in the perimatrix, few (+). These varied within a range of zero (0) to numerous to an abundance (+++/++++) of factor-positive cells. The MMP-9-containing cells were, on average, occasional (0/+) in the matrix and perimatrix, and ranged from zero (0) to few to a moderate (+/++) amount of MMP-9-positive cells (Figure 8b,e). 

TIMP-2-positive cells in the matrix were few (+) in the matrix and occasional (0/+) in the perimatrix, and presented a variance from zero (0) to numerous (+++) factor-positive cells. The mean number of TIMP-4-containing cells in the matrix was moderate to numerous (++/+++), and in the perimatrix, was moderate (++). TIMP-4-positive cells amongst the group subjects ranged from zero (0) to numerous to abundant (+++/++++) (Figure 9b,e). 

In the control group, the mean numbers of MMP-2-positive cells were few (+) in the epithelium and the connective tissue. The relative numbers varied from zero (0) to numerous (+++) in the epithelium, and from an occasional amount (0/+) to few (+) positive cells in the visual field in the connective tissue. On average, MMP-9-positive cells in the epithelium were few (+) and occasional to few (0/+-+) in the connective tissue, ranging from zero (0) to few to a moderate (+/++) amount of factor-containing cells (Figure 8c,f). 

The average number of TIMP-2-positive cells was few to moderate (+/++) in the epithelium, and occasional to few (0/+-+) in the connective tissue, and varied from occasional (0/+) to moderate to numerous (++/+++) in the epithelium and zero (0) to few (+) factor-positive cells in the connective tissue. The TIMP-4-containing cells averaged moderate (++) and few to moderate (+/++) in the epithelium and connective tissue, respectively. The factor-positive cells ranged from few (+) to numerous (+++) within the group (Figure 9c,f). 

### 3.9. Statistical Comparison between the Groups

To determine statistical differences between the children and adult groups, we used the Kruskal–Wallis test. There were no statistically discernible differences between the expressions of tissue factors in the children and adult groups; complete results are shown in Table 3. 

**Table 3:** Kruskal–Wallis test between the children and the adult groups.

Detected Factor		Kruskal–Wallis Test	*p*-Value
Children	Adults		
HβD-2 matrix	HβD-2 matrix	0.587	>0.999
HβD-2 perimatrix	HβD-2 perimatrix	0.480	>0.999
HβD-4 matrix	HβD-4 matrix	0.868	>0.999
HβD-4 perimatrix	HβD-4 perimatrix	−0.719	>0.999
IL-1α matrix	IL-1α matrix	−0.039	>0.999
IL-1α perimatrix	IL-1α perimatrix	0.717	>0.999
IL-10 matrix	IL-10 matrix	0.043	>0.999
IL-10 perimatrix	IL-10 perimatrix	0.868	>0.999
Ki-67 matrix	Ki-67 matrix	0.886	>0.999
Ki-67 perimatrix	Ki-67 perimatrix	0.908	>0.999
NF-κβ matrix	NF-κβ matrix	−0.079	>0.999
NF-κβ perimatrix	NF-κβ perimatrix	0.039	>0.999
VEGF matrix	VEGF matrix	−0.694	>0.999
VEGF perimatrix	VEGF perimatrix	0.486	>0.999
SHH matrix	SHH matrix	0.482	>0.999
SHH perimatrix	SHH perimatrix	0.363	>0.999
MMP-2 matrix	MMP-2 matrix	0.415	>0.999
MMP-2 perimatrix	MMP-2 perimatrix	0.986	0.972
MMP-9 matrix	MMP-9 matrix	−0.365	>0.999
MMP-9 perimatrix	MMP-9 perimatrix	1.290	0.591
TIMP-2 matrix	TIMP-2 matrix	0.576	>0.999
TIMP-2 perimatrix	TIMP-2 perimatrix	0.958	>0.999
TIMP-4 matrix	TIMP-4 matrix	−0.205	>0.999
TIMP-4 perimatrix	TIMP-4 perimatrix	0.159	>0.999

Abbreviations: HβD-2—human beta defensin 2; HβD-4—human beta defensin 4; IL-1α—interleukin 1; IL-10—interleukin 10; Ki-67—proliferation marker; NF-κβ—nuclear factor kappa beta; VEGF—vascular endothelial growth factor; SHH—Sonic hedgehog gene protein; MMP-2—matrix metalloproteinase 2; MMP-9—matrix metalloproteinase 9; TIMP-2—tissue inhibitor of metalloproteinase-2; TIMP-4—tissue inhibitor of metalloproteinase-4.

To evaluate the differences among all of the groups (children, adults, control), we used the Kruskal–Wallis test. We found statistically significant differences and a tendency for statistically significant differences between the relative numbers of HβD-2, HβD-4, Ki-67, NF-κβ, SHH, VEGF, and TIMP-2-containing cells in both cholesteatoma groups, compared with the control group. The complete results are summarized in Table 4. 

**Table 4:** Statistically significant differences between both patient groups and the control group.

Detected Factor	Kruskal–Wallis Test	*p*-Value
Adult HβD-2 P vs. Control HβD-2 CT	2.815	0.015
Children HβD-2 P vs. Control HβD-2 CT	2.498	0.038
Children HβD-4 M vs. Control HβD-4 E	−2.132	0.099 *
Adult Ki-67 M vs. Control Ki-67 E	3.697	0.001
Children Ki-67 M vs. Control Ki-67 E	3.110	0.006
Adult Ki-67 P vs. Control Ki-67 CT	2.577	0.030
Adult NF-κβ M vs. Control NF-κβ E	2.864	0.013
Children NF-κβ M vs. Control NF-κβ E	2.915	0.011
Adult VEGF M vs. Control VEGF E	−2.146	0.096 *
Adult SHH P vs. Control SHH CT	3.146	0.005
Children SHH P vs. Control SHH CT	2.906	0.011
Children TIMP-2 M vs. Control TIMP-2 E	-2.207	0.082 *

Abbreviations: M—matrix; P—perimatrix; E—epithelium; CT—connective tissue; HβD-2—human beta defensin 2; HβD-4—human beta defensin 4; Ki-67—proliferation marker; NF-κβ—nuclear factor kappa beta; VEGF—vascular endothelial growth factor; SHH—Sonic hedgehog gene protein; MMP-9—matrix metalloproteinase 9; TIMP-2—tissue inhibitor of metalloproteinase-2. *—tendency for being statistically significant difference.

### 3.10. Correlations between Tissue Factors in Patient Groups

Most of the correlations were between tissue remodeling factors, pro- and anti-inflammatory factors, and NF-κβ. A summary of similar and statistically significant correlations between cell factors in the children and adult groups is shown in Table 5.

The complete table of correlations in each group (children, adults, and control) are shown in the Supplementary Materials (Tables S2–S4).

**Table 5:** Matching correlating cell factors in the children and adult groups.

Factor 1	Factor 2	Spearman’s Correlation Coefficient; *p* Value	
MMP-2 matrix	MMP-2 perimatrix	Children r = 0.803; *p* = 0.000	Adult r = 0.574; *p* = 0.003
MMP-2 matrix	TIMP-2 matrix	Children r = 0.622; *p* = 0.001	Adult r = 0.484; *p* = 0.014
MMP-2 matrix	SHH matrix	Children r = 0.786; *p* = 0.000	Adult r = 0.719; *p* = 0.000
MMP-2 matrix	NF-κβ matrix	Children r = 0.677; *p* = 0.000	Adult r = 0.399; *p* = 0.048
MMP-2 perimatrix	SHH matrix	Children r = 0.786; *p* = 0.000	Adult r = 0.453; *p* = 0.023
MMP-2 perimatrix	SHH perimatrix	Children r = 0.653; *p* = 0.000	Adult r = 0.460; *p* = 0.021
MMP-9 matrix	IL-1α matrix	Children r = 0.549; *p* = 0.004	Adult r = 0.426; *p* = 0.034
MMP-9 matrix	IL-10 matrix	Children r = 0.418; *p* = 0.038	Adult r = 0.458; *p* = 0.021
MMP-9 perimatrix	TIMP-4 perimatrix	Children r = 0.490; *p* = 0.013	Adult r = 0.664; *p* = 0.000
MMP-9 perimatrix	IL-1α matrix	Children r = 0.642; *p* = 0.001	Adult r = 0.435; *p* = 0.030
MMP-9 perimatrix	IL-1α perimatrix	Children r = 0.714; *p* = 0.000	Adult r = 0.608; *p* = 0.001
MMP-9 perimatrix	IL-10 perimatrix	Children r = 0.468; *p* = 0.018	Adult r = 0.601; *p* = 0.001
MMP-9 perimatrix	NF-κβ perimatrix	Children r = 0.614; *p* = 0.001	Adult r = 0.790; *p* = 0.000
MMP-9 perimatrix	Ki-67 perimatrix	Children r = 0.624; *p* = 0.001	Adult r = 0.677; *p* = 0.000
MMP-9 perimatrix	HβD-2 matrix	Children r = 0.487; *p* = 0.014	Adult r = 0.464; *p* = 0.019
TIMP-2 matrix	TIMP-2 perimatrix	Children r = 0.685; *p* = 0.000	Adult r = 0.676; *p* = 0.000
TIMP-2 matrix	SHH matrix	Children r = 0.537; *p* = 0.006	Adult r = 0.478; *p* = 0.016
TIMP-2 matrix	NF-κβ matrix	Children r = 0.504; *p* = 0.010	Adult r = 0.473; *p* = 0.017
TIMP-2 matrix	HβD-2 matrix	Children r = 0.505; *p* = 0.010	Adult r = 0.416; *p* = 0.038
TIMP-2 perimatrix	IL-1α matrix	Children r = 0.457; *p* = 0.022	Adult r = 0.505; *p* = 0.010
TIMP-2 perimatrix	IL-10 matrix	Children r = 0.423; *p* = 0.035	Adult r = 0.630; *p* = 0.001
TIMP-4 matrix	TIMP-4 perimatrix	Children r = 0.841; *p* = 0.000	Adult r = 0.431; *p* = 0.031
TIMP-4 matrix	SHH matrix	Children r = 0.681; *p* = 0.000	Adult r = 0.436; *p* = 0.029
TIMP-4 matrix	NF-κβ matrix	Children r = 0.738; *p* = 0.000	Adult r = 0.540; *p* = 0.005
TIMP-4 perimatrix	IL-1α matrix	Children r = 0.641; *p* = 0.001	Adult r = 0.457; *p* = 0.022
TIMP-4 perimatrix	IL-1α perimatrix	Children r = 0.663; *p* = 0.000	Adult r = 0.637; *p* = 0.001
TIMP-4 perimatrix	IL-10 matrix	Children r = 0.638; *p* = 0.001	Adult r = 0.425; *p* = 0.034
TIMP-4 perimatrix	IL-10 perimatrix	Children r = 0.721; *p* = 0.000	Adult r = 0.638; *p* = 0.001
TIMP-4 perimatrix	NF-κβ matrix	Children r = 0.654; *p* = 0.000	Adult r = 0.422; *p* = 0.036
TIMP-4 perimatrix	NF-κβ perimatrix	Children r = 0.457; *p* = 0.022	Adult r = 0.749; *p* = 0.000
TIMP-4 perimatrix	Ki-67 matrix	Children r = 0.481; *p* = 0.015	Adult r = 0.402; *p* = 0.046
TIMP-4 perimatrix	Ki-67 perimatrix	Children r = 0.414; *p* = 0.040	Adult r = 0.651; *p* = 0.000
TIMP-4 perimatrix	HβD-2 matrix	Children r = 0.568; *p* = 0.003	Adult r = 0.404; *p* = 0.045
TIMP-4 perimatrix	HβD-2 perimatrix	Children r = 0.397; *p* = 0.049	Adult r = 0.545; *p* = 0.005
SHH matrix	NF-κβ matrix	Children r = 0.753; *p* = 0.000	Adult r = 0.549; *p* = 0.005
SHH perimatrix	Ki-67 matrix	Children r = 0.746; *p* = 0.000	Adult r = 0.646; *p* = 0.000
SHH perimatrix	HβD-2 perimatrix	Children r = 0.428; *p* = 0.033	Adult r = 0.527; *p* = 0.007
IL-1α matrix	IL-1α perimatrix	Children r = 0.716; *p* = 0.000	Adult r = 0.557; *p* = 0.004
IL-1α matrix	IL-10 matrix	Children r = 0.709; *p* = 0.000	Adult r = 0.813; *p* = 0.000
IL-1α matrix	IL-10 perimatrix	Children r = 0.720; *p* = 0.000	Adult r = 0.762; *p* = 0.000
IL-1α matrix	NF-κβ perimatrix	Children r = 0.406; *p* = 0.044	Adult r = 0.519; *p* = 0.008
IL-1α matrix	HβD-2 matrix	Children r = 0.700; *p* = 0.000	Adult r = 0.827; *p* = 0.000
IL-1α perimatrix	IL-10 perimatrix	Children r = 0.694; *p* = 0.000	Adult r = 0.640; *p* = 0.001
IL-1α perimatrix	NF-κβ perimatrix	Children r = 0.510; *p* = 0.009	Adult r = 0.692; *p* = 0.000
IL-1α perimatrix	Ki-67 perimatrix	Children r = 0.441; *p* = 0.027	Adult r = 0.583; *p* = 0.002
IL-1α perimatrix	HβD-2 matrix	Children r = 0.630; *p* = 0.001	Adult r = 0.499; *p* = 0.011
IL-10 matrix	IL-10 perimatrix	Children r = 0.668; *p* = 0.000	Adult r = 0.801; *p* = 0.000
IL-10 matrix	NF-κβ perimatrix	Children r = 0.536; *p* = 0.006	Adult r = 0.554; *p* = 0.004
IL-10 matrix	VEGF matrix	Children r = 0.559; *p* = 0.004	Adult r = 0.611; *p* = 0.001
IL-10 matrix	HβD-2 matrix	Children r = 0.828; *p* = 0.000	Adult r = 0.841; *p* = 0.000
IL-10 matrix	HβD-2 perimatrix	Children r = 0.677; *p* = 0.000	Adult r = 0.462; *p* = 0.020
IL-10 perimatrix	NF-κβ matrix	Children r = 0.602; *p* = 0.001	Adult r = 0.396; *p* = 0.050
IL-10 perimatrix	VEGF matrix	Children r = 0.696; *p* = 0.000	Adult r = 0.687; *p* = 0.000
IL-10 perimatrix	HβD-2 matrix	Children r = 0.592; *p* = 0.002	Adult r = 0.686; *p* = 0.000
IL-10 perimatrix	HβD-2 perimatrix	Children r = 0.516; *p* = 0.008	Adult r = 0.687; *p* = 0.000
NF-κβ matrix	VEGF matrix	Children r = 0.595; *p* = 0.002	Adult r = 0.414; *p* = 0.039
NF-κβ matrix	HβD-2 matrix	Children r = 0.750; *p* = 0.000	Adult r = 0.418; *p* = 0.038
NF-κβ perimatrix	Ki-67 perimatrix	Children r = 0.494; *p* = 0.012	Adult r = 0.571; *p* = 0.003
NF-κβ perimatrix	VEGF matrix	Children r = 0.637; *p* = 0.001	Adult r = 0.474; *p* = 0.017
NF-κβ perimatrix	HβD-2 matrix	Children r = 0.621; *p* = 0.001	Adult r = 0.526; *p* = 0.007
NF-κβ perimatrix	HβD-2 perimatrix	Children r = 0.635; *p* = 0.001	Adult r = 0.748; *p* = 0.000
Ki-67 matrix	HβD-2 perimatrix	Children r = 0.520; *p* = 0.008	Adult r = 0.498; *p* = 0.011
VEGF matrix	VEGF perimatrix	Children r = 0.745; *p* = 0.000	Adult r = 0.429; *p* = 0.032
VEGF matrix	HβD-2 perimatrix	Children r = 0.509; *p* = 0.009	Adult r = 0.413; *p* = 0.040
HβD-2 matrix	HβD-2 perimatrix	Children r = 0.748; *p* = 0.000	Adult r = 0.477; *p* = 0.016

Abbreviations: HβD-2—human beta defensin 2; IL-1α—interleukin 1; IL-10—interleukin 10; Ki-67—proliferation marker; NF-κβ—nuclear factor kappa beta; VEGF—vascular endothelial growth factor; SHH—Sonic hedgehog gene protein; MMP-2—matrix metalloproteinase 2; MMP-9—matrix metalloproteinase 9; TIMP-2—tissue inhibitor of metalloproteinase-2; TIMP-4—tissue inhibitor of metalloproteinase-4; r—Spearman’s correlation coefficient; *p*—*p*-value; r = 0.2–0.4 a weak correlation, r = 0.4–0.6 a moderate correlation, r = 0.6–0.8 a strong correlation and r = 0.8–1.0 a very strong correlation.

## 4. Discussion

### 4.1. Human Beta Defensins

Our study did not find statistically discernible differences in the relative numbers of HβD-2- and HβD-4-positive cells (in the matrix and perimatrix) between the adult and the children groups. We could not find similar studies in which these factors were compared between both groups by other authors.

However, we found that the adult and children groups both had statistically significant over-expressions of the relative numbers of HβD-2 in cholesteatoma compared with the control group. Similar to that observed by other authors, we found up-regulation of HβD-2 in cholesteatoma compared with control skin [9,10]. Interestingly, we found that HβD-4 is less expressed in cholesteatoma tissue compared with control skin; however, only the children group showed less of a tendency to be statistically discernible than the relative amount of HβD-4 in the control group. 

In addition to previous findings, we found several important and, most importantly, similar correlations between HβD-2 and other cell factors in both the adult and children groups, including HβD-2 intercorrelations with IL-1α and intercorrelation between IL-1α and NF-κβ. These findings support the discoveries of other authors that IL-1α stimulates the production of HβD-2 and that NF-κβ is a regulator of this process in human cholesteatoma [47,48]. Furthermore, we found an intercorrelation between HβD-2 and IL-10 in the perimatrix, supporting the data that Kanda et al. [49] presented regarding the HβD-2 stimulation of IL-10 in T-cells. In addition, this may suggest cooperation between IL-10 and HβD-2 in anti-inflammatory activity in cholesteatoma. Unlike HβD-2, we did not find similar correlations with HβD-4, and the relative number of HβD-4 in cholesteatoma was lower than HβD-2. This could suggest that the main defensin against invading pathogens in children and adult cholesteatoma is HβD-2, or that the activity of HβD-4 is suppressed. However, further studies should be conducted to understand this better. 

### 4.2. Pro- and Anti-Inflammatory Cytokines

We did not find statistically significant differences between the pro- and anti-inflammatory tissue factors when comparing the adult and children groups. However, there were limited data available in databases to support our findings. Additionally, we did not find statistically significant differences when comparing both groups with the control group, though IL-1α levels in the children and adult groups were higher compared with control skin. These findings are supported by other authors who also found similar statistically significant differences between control skin and cholesteatoma tissue [21,50]. We found strong positive correlations between IL-1α and IL-10 in the adult and children groups; these findings suggest that pro- and anti-inflammatory actions are not dependent on age, and that pro-inflammatory cytokine changes in the cholesteatoma are strongly related to an anti-inflammatory factor action in the same tissue. 

In contrast with the strong positive correlation between IL-1α and IL-10 in both groups of cholesteatoma, we found a very strong but negative correlation between IL-1α and IL-10 in the control group (Supplementary File S2). This finding indicates an idea that there is a dysregulation between IL-1α and IL-10 in cholesteatoma tissue, which may be one of the reasons cholesteatomas cause chronic inflammation and induce destruction in the middle ear; similar conclusions were drawn by Kuczkowski et al. [21]. 

Furthermore, IL-1α induces osteoclast function and increases bone matrix degradation [51], and it is known that MMP-2 and MMP-9 also cause bone matrix degradation for patients with cholesteatoma [36,37]. Another study proved that IL-1α acts as an inducer of MMP-2 and MMP-9, which then promote osteoclast production and activity [52]. The intercorrelations we found among IL-1α and MMP-2 and MMP-9 may support the theory that MMP activity is also regulated by IL-1α in cholesteatoma tissue. 

As an anti-inflammatory cytokine, IL-10 has been shown to suppress MMP-9 [53], but induce TIMP-2 and TIMP-1 [53,54]. However, these studies prove this action in non-cholesteatoma tissue. Our study showed similar intercorrelations between MMP-9, TIMP-2, TIMP-4, and IL-10 in the adult and children groups. These findings suggest that IL-10 may also regulate the activities of MMPs and TIMPs in cholesteatoma. We can even speculate further, that the dysregulation between IL-1α and IL-10 could affect the balance of MMPs and TIMPs and result in bone remodulation in the middle ear.

### 4.3. Proliferation Marker Ki-67

In the present study, we did not find statistically discernible differences between the relative numbers of Ki-67-positive cells when comparing pediatric and adult cholesteatoma, which is similar to the data presented by Sikka et al. [55]. These findings show that cell proliferation in cholesteatoma does not depend on patient age. In contrast with our results, Bujía et al. [56] have showed an increased proliferation index in the pediatric group compared with adults. The larger and more equal study group size in our research may be one of the reasons why there are differences between the studies; another reason may be that Bujía et al. used antibodies in frozen tissue sections, rather than being paraffin-embedded, which they claim as a disadvantage.

Our results show a statistically significant increase in the number of Ki-67-containing cells in the children and adult groups compared with control skin and are similar to the data presented by other authors [57,58]. These findings show that Ki-67 can be used as a reliable marker to detect proliferation in the cholesteatoma, in addition to the hyperproliferative activity of cholesteatoma cells when compared with unchanged skin cells. 

### 4.4. Transcription Factor NF-κβ

NF-κβ is present in almost every activity in human organisms [26]. It mainly regulates inflammatory processes, cell proliferation, and apoptosis [14,27]. Our data do not show statistically discernible differences between the adult and children groups. This finding suggests that the inflammatory and cell proliferation processes do not depend on the age of the patient. However, we found a statistically discernible over-expression of NF-κβ in the children and adult groups compared with control skin. These findings are supported by Byun et al. [28], who presented the up-regulation of NF-κβ in cholesteatoma compared with retroauricular skin. 

It is known that NF-κβ acts through a pathway with Ki-67 (the inhibitor of the DNA binding protein 1 (Id1) → NF-κB → cyclin D1 → Ki-67) and activates cell proliferation in human cholesteatoma [59]. Our study supports these findings by showing moderate correlation between NF-κβ and Ki-67 in the adult and children groups. Further, we found a strong correlation between NF-κβ and MMP-2 and MMP-9 in both groups of patients. It has been mentioned that NF-κβ can stimulate MMP production and tumor growth, especially angiogenesis [60]. Next, we found moderate and strong correlations between NF-κβ and VEGF in both groups, data which suggest that NF-κβ induces VEGF and neo-angiogenesis in cholesteatoma tissue; this is supported by Fukudome et al. [29], who proved that NF-κβ promotes VEGF activation. Furthermore, it is known that VEGF, MMP-2, and MMP-9 affect each other to promote or inhibit angiogenesis in different tumors [61], and that the intercorrelations we found in our research between MMP-2, MMP-9, and VEGF may support the belief that similar activities are present in cholesteatoma tissue; however, this needs to be proved. 

NF-κβ participates in every process we have reviewed in this research (defense against infection, inflammation, proliferation, angiogenesis, tissue remodulation) and intercorrelates with every tissue factor we observed. Once again, this shows the complexity of NF-κβ as a transcription factor.

### 4.5. Angiogenetic Factor

VEGF is one of the most potent angiogenetic factors that has been proven to cause neo-angiogenesis in human cholesteatoma [29,30]. Our data do not reveal statistically significant differences between the children and adult groups. However, we found a tendency for a statistically significant decrease in VEGF-positive cells (in the matrix) in the adult group compared with the control group (epithelium), but not between the children and control groups, though the relative numbers also decreased in the children group. However, the authors of this study understand that the formation of new blood vessels is much greater in cholesteatoma compared with unchanged skin [62]. Our results can be explained through different epithelial tumors presenting different amounts of VEGF; for example, basal cell carcinomas were weakly stained for VEGF, in addition to the fact that, in some lesions, VEGF may be released more extracellularly than intracellularly [63]. It has been proven that cholesteatoma keratinocytes release VEGF into the perimatrix and induce angiogenesis in a paracrine manner [29]. These findings are supported by our data, from which we found a strong and moderate correlation between VEGF in the matrix and VEGF in the perimatrix. Furthermore, to explain why VEGF-containing cells were not statistically significantly greater in the perimatrix than in the control skin connective tissue, we examined a relative amount and compared positive and negative endotheliocytes in the visual field, not the absolute number of VEGF-containing cells.

### 4.6. Sonic Hedgehog

We did not find statistically significant differences in the expression of the SHH gene protein between the adult and children groups, but there was statistically significant overexpression of SHH-positive cells in the adult and children groups compared with the control group. Our study suggests that the SHH gene plays a role mainly in the development of human cholesteatoma, and it is not age dependent. However, because of the limited information available on SHH’s role in cholesteatoma, targeted studies should be carried out to gather more evidence about its role in cholesteatoma pathogenesis. Nevertheless, in the oncology, it has been proven that the SHH pathway has a part in the growth of different tumors [64]. 

### 4.7. Remodeling Factors

Remodeling factors are one of the tissue factors responsible for bone degradation and cholesteatoma aggressiveness in the middle ear [36,37]. In contrast with Dornelles et al. [65], whose study showed that MMP-2 and MMP-9 are overexpressed in pediatric cholesteatoma compared with adult cholesteatoma, we did not find statistically significant differences in the relative amounts of MMP-2-, MMP-9-, TIMP-2-, and TIMP-4-positive cells between the children and adult groups. Our data suggest that the tissue remodulation achieved by matrix metalloproteinases and their inhibitors is the same regardless of the age of the patient. 

Furthermore, our data reveal the under-expression of MMP-9-containing cells in the adult and children groups compared with the control group, but it did not reach a statistically discernible difference. However, the numbers of MMP-2-positive cells increased in both groups of patients compared with the control group. These results are in an agreement with Banerjee et al. [66], who showed no up-regulation of MMP-2 and MMP-9 compared with deep meatal skin, and with Rezende et al. [67], whose study demonstrated that MMP-9 detected with the PCR method was negative in cholesteatoma samples. In addition, our study revealed that many samples of cholesteatoma were also negative or weakly stained for MMP-9 immunohistochemically. Next, we demonstrated that the relative number of TIMP-2-containing cells showed a tendency for being statistically significantly decreased in the children’s group compared with the control group. In the adult group, we also observed a reduction compared with the control group in TIMP-2-positive cells. Our data on TIMP-2 are similar to the data from Kaya et al. [68], who showed a decrease in TIMP-2 in cholesteatoma compared with postauricular skin. We did not find statistically discernible differences between TIMP-4 in the patient groups versus the control group, but it was increased in the patient groups. Additionally, TIMP-4 was significantly more expressed in cholesteatoma tissue than TIMP-2. Based on our study, we suggest that in cholesteatoma tissue there is an imbalance between MMPs and TIMPs that may result in tissue remodulation in the middle ear. We substantiate this by the increased amount of MMP-2 and decreased TIMP-2 in the cholesteatoma groups compared with control skin, as well as positive correlations between MMPs and TIMPs in the cholesteatoma tissue. This theory is also supported by Schönermark et al. [44], who explain that the imbalance between MMPs and TIMPs causes proteolysis in the extracellular matrix, and that this can cause bone remodulation in affected patients. As our results of the relative numbers of MMPs and TIMPs and their intercorrelations are similar in the children and adult groups, it means that the explained processes should also be similar in both groups of patients. 

Furthermore, in the previous sections, we described how NF-κβ affects or is affected by cytokines and MMPs. In our study, we found a positive correlation between NF-κβ and TIMP-2 in adult and pediatric cholesteatoma. To explain this correlation, we searched the literature and found that TIMP-2 can directly regulate NF-κβ and prevent cells from apoptosis [69]; thus, we could speculate that TIMP-2 may also affect NF-κβ and induce cell proliferation in cholesteatoma tissue. 

In summary, our results prove that, histopathologically speaking, adult and children cholesteatomas do not differ. These results are supported by Sikka et al. [55]. However, other studies like those of Dornelles et al. [66] and Bujía et al. [56] showed an increase in MMPs and proliferation index in the children group. We do not deny that there may be differences between children and adults clinically, but this is due to non-histopathologic reasons, such as more frequent acute middle ear infections in children, differentiation of mastoid cell aeration, anatomy of the eustachian tube, and hormonal changes, all of which could affect progress of the disease [70]. 

The novelty of our study is that we used twelve different tissue factors, which, to our knowledge, is the largest number of factors in one study used to describe child and adult cholesteatoma. The advantage of our study is that we evaluated cholesteatoma tissue from different aspects, such as inflammation, tissue remodulation, angiogenesis, and cell proliferation. Additionally, we studied previously unused cell factors, such as HβD-4, SHH, and TIMP-4. Furthermore, we demonstrated the complexity of the pathophysiology of cholesteatoma by showing and explaining many intercorrelations between the studied cell factors. We observed that almost every cell factor intercorrelates with another and found explanations for each of these intercorrelations in other studies. 

However, we are aware of the limitations of our study. The immunohistochemistry and semi-quantitative evaluation method is a valid and widely used technique with which to evaluate tissue, but it cannot evaluate the concentrations of studied proteins in the cholesteatoma tissue; methods like the enzyme-linked immunosorbent assay (ELISA) or Western blot would be beneficial for IHC-stained samples. Another limitation is the relatively small control group, and the fact that material was obtained from cadavers; however, ethical considerations required the use of this control group. 

## 5. Conclusions

There are no differences in tissue factors regulating defense against invading pathogens, pro- and anti-inflammatory action, proliferation, angiogenesis, remodulation, and the regulation of these actions between adult and pediatric cholesteatoma. 

From defensins, HβD-2 is more active and involved in inflammatory processes compared with HβD-4, regardless of patient age. 

Ki-67 is a reliable non-age-dependent proliferation marker of cholesteatoma.

The dysregulation between pro- and anti-inflammatory cytokines is one of the key points in cholesteatoma pathogenesis for both adults and children.

The over-expression of NF-κβ in cholesteatoma tissue compared with unchanged skin and intercorrelations with different tissue factors highlight its important role in cell proliferation, inflammation, and tissue remodulation processes. 

The increased expression of the SHH gene protein in the involvement of the perimatrix indicates endodermal gene involvement in the expansion of cholesteatoma.

The decreased number of TIMP-2 and increased MMP-2 in patient groups compared with the control group are the cause of the intensive remodulation in patients with cholesteatomas.

## Figures and Tables

**Table 1:** Explanation of semi-quantitative method.

Grading Scale	Explanation of Grading Scale	Percentage of Factor-Positive Cells in the Visual Field
0	No positive structures	0%
0/+	Occasional positive structures	12.5%
+	Few positive structures	25%
+/++	Few-to-moderate number of positive structures	37.5%
++	Moderate number of positive structures	50%
++/+++	Moderate-to-numerous positive structures	62.5%
+++	Numerous positive structures	75%
+++/++++	Numerous-to-abundant structures	87.5%
++++	An abundance of positive structures in the visual field	100%

## Data Availability

Data supporting the results can be found in the Supplementary Materials files (Figures S1 and S2. Tables S1–S4).

## Supplementary Materials

The following supporting information can be downloaded at: https://www.mdpi.com/article/10.3390/diagnostics14060662/s1. Figure S1: Negative control. Figure S2: Pictures of each criterion of semi-quantitative method. Table S1: Complete table of relative tissue factor values in each group. Table S2: Adult group correlations.
