# Supplementary material for: Comparison of Tissue Factors in the Ontogenetic Aspects of Human Cholesteatoma
Source: Diagnostics (Basel). 2024 Mar 21;14(6):662. doi: 10.3390/diagnostics14060662 (PMC10969033; doi:10.3390/diagnostics14060662)
Supplement: Supplementary file 1 [file diagnostics-14-00662-s001.zip › diagnostics-2842272-supplementary.pdf]

Supplementary file

Figure S1 Negative control.

The example of test sample of the cholesteatoma: MMP2 negative control in cholesteatoma tissue. X 250.

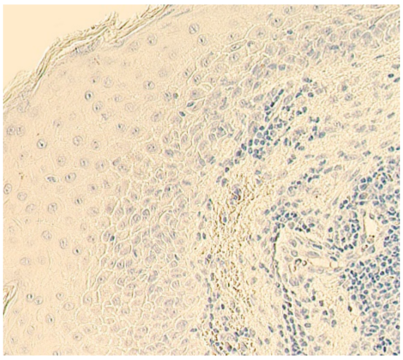

Positive controls in accordance with the manufacturer's guidelines and negative controls with exclusion of primary antibody were developed.

Figure S2 Pictures of each criterion of semi-quantitative method.

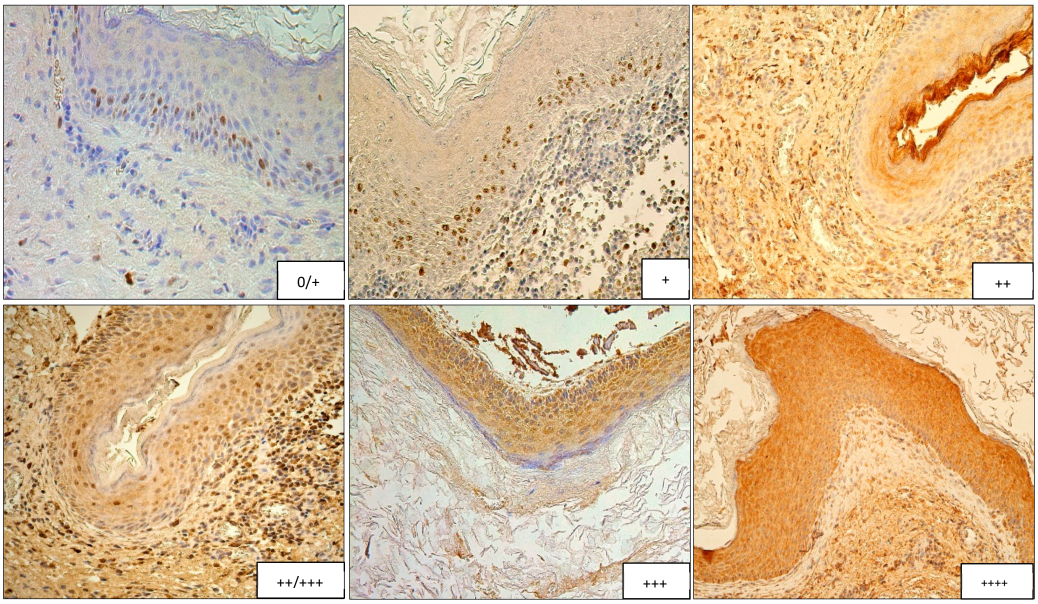

0/+ = occasional positive structures; **+** = few positive structures; **++** = moderate number of positive structures; **++/+++** = moderate to numerous positive structures; **+++** = numerous positive structures; **++++** = an abundance of positive structures in the visual field.

Table S1

Complete table of relative tissue factor values in each group

1. Children group

	Gender	Age	Ki-67 M	Ki-67 P	MMP-2 M	MMP-2 P	TIMP-2 M	TIMP-2 P	IL-1 M	IL-1 P	IL-10 M	IL-10 P	HBD-2 M	HBD-2 P	HBD-4 M	HBD-4 P	Shh M	Shh P	MMP-9M	MMP-9P	TIMP-4M	TIMP-4P	NFKB M	NFKB P	VEGF M	VEGF P
1	M	11	+	+	+/++	+	+/++	0/+	+++	+	+++	++	++/+++	++	+/++	0/+	++/+++	++	0/+	0/+	++/+++	++	++/+++	++	++/+++	0/+
2	M	16	+	0/+	+	0/+	0	0/+	+	+	+++	+/++	++	+/++	0/+	+	++/+++	++	0/+	0-0/+	++/+++	++/+++	++	+	++	0
3	F	9	0	0	0	0	0	0	0	0/+	0	0/+	0	0	0	0/+	0	0	0	0	+	+/++	0/+	0/+	++	0
4	M	17	0/+	0-0/+	0/+	0/+	0	0	+	0/+	++	+	+/++	+	0	+	+/++	+	0	0-0/+	++	++	++	+/++	++	0/+
5	M	10	0-0/+	0-0/+	++	++	0	0/+	++	+	+/++	++	+/++	0/+	0	0/+	++	+/++	0/+	0/+	+++/++++	+++/++++	++/+++	+	+++	++
6	F	16	+	0/+	+/++	+/++	+	0/+	++	+/++	++	+/++	++	+	0	0/+	+++	++/+++	++	+/++	+++/++++	+++	+++	+/++	+++/++++	++/+++
7	F	16	0/+	0-0/+	+	+	+/++	0/+	++	+/++	++	++	++	+/++	0	0-0/+	++/+++	++	++	0/+	+++/++++	+++	++	+/++	+++	++
8	M	13	++	0/+	+/++	++	0/+	0/+	+++	++	+++	++	++	++	0-0/+	0/+	+++	++	++	+/++	+++/++++	+++	+++	++	+++	++
9	F	12	0-0/+	0	++	+/++	+/++	0-0/+	++/+++	+	++/+++	+	++	+/++	0	0-0/+	++	+	0	0	++/+++	++	++/+++	0/+	++	0/+
10	F	15	0-0/+	0-0/+	+/++	+	0-0/+	0/+	+	0/+	++	+/++	++	++	0	0/+	++/+++	+/++	0	0-0/+	+++/++++	++	++	0/+	++	+
11	F	15	0	0	+	0/+	0	0	0	0/+	0/+	0/+	+	0/+	0	0/+	+	0/+	0	0-0/+	+/++	+	0	0	+	0
12	M	15	0/+	0-0/+	+/++	0/+	0/+	0	++	0/+	+/++	+	+	0/+	0	0	++	+/++	+	0/+	++/+++	++	++	0/+	++	0
13	M	6	0-0/+	0-0/+	0/+	0/+	0/+	0/+	0/+	+/++	0/+	0/+	+/++	0/+	0-0/+	0/+	+/++	+	+	+	+/++	+	+/++	+/++	++/+++	+
14	F	16	0/+	0	++/+++	+++	+++/++++	+/++	0	0	+/++	0	+/++	+/++	+	0/+	+++	++	0/+	0-0/+	0	0	++	++	++	0
15	F	8	0/+	0-0/+	++	++	0	0	0	0/+	+/++	0-0/+	+/++	+	0	0-0/+	+++	++	+	0-0/+	+++/++++	++/+++	++	++	++	0/+
16	M	6	0/+	0-0/+	++/+++	++/+++	+++/++++	++	+/++	+	++	+	+	0/+	0/+	0-0/+	+++	++	0	0/+	+++	++/+++	++	+/++	++	0/+
17	F	5	0	0-0/+	0	0	0	0	0	0-0/+	0	0	0	0	0-0/+	0-0/+	+	+/++	0	0	+/++	+/++	0	0	0	0
18	M	5	0/+-+	0/+	+/++	+/++	0	0	0/+	0/+	0	0/+	0/+	0/+	++/+++	+/++	+++	+++	0	0-0/+	++/+++	+/++	++	0/+	0/+	0-0/+
19	M	15	0	0	0/+	0/+	0	0-0/+	0	0/+	0	0-0/+	0	0/+	0/+	0-0/+	+/++	+	0	0-0/+	+/++	+/++	0-0/+	0-0/+	0	0
20	M	17	+	0/+	++	+/++	+	+	++	0/+	0/+	0/+	+/++	0/+	+/++	0/+	+++	++/+++	0/+	0/+	+++	+/++	++/+++	+	+	0-0/+
21	M	14	+/++	+/++	+++	++/+++	++	0/+	+++	++/+++	++/+++	+	++	+	+++/++++	+++	+++/++++	++/+++	+/++	++	+++/++++	++/+++	+++	++	+/++	0/+
22	F	13	0-0/+	0	0/+	0/+	0/+	0/+	+/++	+	+/++	++	+	0/+	0/+	0/+	++	++	0/+	0-0/+	++	++	++	0/+	++	+
23	M	14	0-0/+	0	+/++	0/+	+	0-0/+	+/++	0/+	+	0/+	+/++	0-0/+	+	0/+	++	+	+	0-0/+	++	++	+	0-0/+	+	0/+
24	M	17	0-0/+	0-0/+	+	0/+	0/+	0	+	0/+	++	0/+	+	0-0/+	0/+	0-0/+	+	0/+	0/+	0-0/+	+/++	+/++	0/+	0	+	0
25	M	17	0/+	0-0/+	++/+++	0/+	++	+	+++	+/++	+++	+	++/+++	0/+	++	0/+	+++	+	++	0/+	+++	++/+++	+++	+/++	++/+++	+

2. Adult group

	Gender	Age	Ki-67 M	Ki-67 P	MMP-2 M	MMP-2 P	TIMP-2 M	TIMP-2 P	IL-1 M	IL-1 P	IL-10 M	IL-10 P	HBD-2 M	HBD-2 P	HBD-4 M	HBD-4 P	Shh M	Shh P	MMP-9M	MMP-9P	TIMP-4M	TIMP-4P	NFKB M	NFKB P	VEGF M	VEGF P
1	F	58	+	+	++/+++	+/++	+++	+	+/++	+	++/+++	++	++/+++	++	++	+	+++	++	0	0/++	+/++	+++	++	+++	++/+++	0/+
2	M	46	0/+	0-0/+	+/++	+/++	0	0	+/++	+	+	+	++	++	0	++	++/+++	++	0/+	0/+	+/++	+/++	+/++	+	+	0
3	M	23	0-0/+	0-0/+	0	+	0/+	+/++	++/+++	+++	+++	+++	++	+/++	+	0/+	++	++	0/+	0/+	++/+++	++/+++	++	++	+/++	0/+
4	M	38	+	0/+-+	+/++	0/+	0/+	0/+	+++	+/++	+++	++	++/+++	++	+/++	+	++/+++	++	0/+	0/+	++/+++	++/+++	+++	+++	++/+++	0
5	F	75	0/+	0/+	0/+	+/++	0	0/+	++	+/++	++	++	++	+/++	0/+	0/+	++	+/++	0	0/+-+	++/+++	++	++	++	++	0
6	F	28	0/+	+/++	0/+	+	0-0/+	0-0/+	++	++	+/++	++	+/++	+	0	0-0/+	+/++	++	+	+	+++	+++	++	++	+++	++
7	F	31	+	0-0/+	+	0/+	0-0/+	0-0/+	0/+	0/+	+/++	+	+	+	0-0/+	0-0/+	++	++	+/++	0/+-+	+++/++++	+++	++/+++	++	++/+++	0/+
8	M	38	0/+	0/+	+/++	+	+/++	0/+	+/++	+	++/+++	+/++	++	+	0	0-0/+	+++	+	0	0/+	++/+++	++	++	0/+	++/+++	+/++
9	M	26	+	+	+	+/++	0/+	0/+	+/++	++	+/++	+/++	+/++	+/++	0	0-0/+	++	++/+++	0/+	+	++/+++	+++	+	++	+	+
10	M	39	0/+	0	0-0/+	0-0/+	0	0	0/+	0-0/+	0/+	0/+	+	0	0	0	+	0/+	0	0	++	+/++	+	0	+/++	0
11	F	22	0-0/+	0/+	++	++	++	++	++	+/++	++/+++	++	++/+++	+	0	0-0/+	++/+++	+/++	+	+	++/+++	++	+++	++	++/+++	+/++
12	M	19	0-0/+	0	+/++	+/++	++	++	++	+	++/+++	++	+/++	+	0	0-0/+	+++	+	+	0/+	+++	++	++	+/++	++/+++	++
13	F	45	0/+	0	++	++	0	0	0	0	0/+	0/+	0/+	0/+	0	0	+/++	++	0	0-0/+	0	0	+	0	+/++	0
14	F	24	+	0	++	+/++	0	0-0/+	0	0	+	0/+	+	+	+	+	+++	++/+++	0	0	++/+++	+/++	+/++	0/+	++/+++	0/+
15	M	39	0/+-+	0-0/+	++/+++	+/++	0/+	0-0/+	0/+	+/++	0	0/+	+	0-0/+	++	0/+	+++	++	0	0-0/+	+++	++	++/+++	0/+	0-0/+	0-0/+
16	F	40	0-0/+	0/+	++	+/++	+/++	0-0/+	+/++	0/+	0/+	0-0/+	+/++	0-0/+	++	0/+	+++	+/++	0	0/+	+++	+/++	++	0-0/+	0-0/+	0/+
17	F	27	+/++	+/++	+++/++++	+++	++/+++	0/+	0	+	0	+	+	++	+++	+/++	++++	+++	0	0/+	+++/++++	++	+++	0/+	+/++	+/++
18	F	41	0/+	0-0/+	++	+	+	0-0/+	+/++	0-0/+	+	0-0/+	+/++	0/+	+/++	0-0/+	++/+++	++	0/+	0-0/+	+++	++	++	0-0/+	0/+	0-0/+
19	F	32	0-0/+	0/+	+/++	+	0	0-0/+	0	+/++	0	0	+	0/+	0	0-0/+	+++	+/++	0/+	0/+	+++	++	+/++	+	0-0/+	0/+
20	M	41	0/+	0-0/+	++/+++	+/++	++	0/+	++	+	+++	+/++	++	0/+	0-0/+	0-0/+	+++	++	+/++	0-0/+	++/+++	++	++/+++	0/+	++/+++	+
21	F	74	0/+-+	0/+	+/++	+	+	0/+	++	+	++	+/++	++	+	0/+	0/+	++	+	+/++	0/+	+++	++/+++	+/++	0/+	+/++	0/+
22	M	26	0-0/+	0	+/++	0/+	+/++	+	+/++	+	++/+++	+	++	0/+	+/++	0/+	++/+++	+/++	+/++	0/+	++/+++	++	++/+++	0/+	++	+/++
23	M	50	0-0/+	0	0/+	0/+	+	+	0/+	0/+	+	0/+	+	0	0/+	0-0/+	+/++	0/+	0-0/+	0	+	+/++	+	0	+	0/+
24	F	70	0/+	0	+/++	0/+	0/+-+	0/+	+/++	0/+	+/++	0/+	+/++	0-0/+	+/++	0/+	+/++	0/+	+	0/+	+/++	+/++	+	0/+	+	0
25	F	34	0-0/+	0/+	+	+/++	0/+	0/+	+	0/+	+/++	+	+	+	++	0/+	++	+/++	+	0/+	+/++	++	+/++	+	+/++	+

3. Control group

			Ki-67 M	Ki-67 P	MMP-2 M	MMP-2 P	TIMP-2 M	TIMP-2 P	IL-1 M	IL-1 P	IL-10 M	IL-10 P	HBD-2 M	HBD-2 P	HBD-4 M	HBD-4 P	Shh M	Shh P	MMP-9M	MMP-9P	TIMP-4M	TIMP-4P	NFKB M	NFKB P	VEGF M	VEGF P
		Age	E	CT	E	CT	E	CT	E	CT	E	CT	E	CT	E	CT	E	CT	E	CT	E	CT	E	CT	E	CT
1		-	0	0	0/+	+	0/+	+	+	0/+	+	+	+	0/+	+	0/+	0	0	0/+	0/+	++	+	0	0-0/+	++/+++	+
2		-	0	0	0	+	+	+	0	+	++	++	+	0	+	0/+	++	+	+/++	+	++/+++	++	0/+	0/+	++/+++	+
3		-	0	0	0	0/+	+	0	0/+	+	++	++	0-0/+	0	+/++	+	0/+	0	0/+	0/+	+/++	+/++	0	0	++	0/+
4		-	0-0/+	0-0/+	+++	+	++/+++	+	0/+	0/+	++/+++	++	++	0/+	+	0/+	+++/++++	+	+/++	0-0/+	++/+++	++	+	0/+	+++	+/++
5		-	0	0	++/+++	+	++/+++	+	0	0/+	++/+++	++	++	0/+	++	+	+++/++++	+	+/++	+/++	+++	++	++	+/++	+++	+
6		-	0-0/+	0-0/+	+	+	++	0/+	+	0/+	+	0/+	0/+	0	+	0/+	+/++	0/+	+	0/+	++/+++	++	+	0/+	++	0/+
7		-	0	0	0/+	+	+/++	0/+	+/++	+	+/++	+	0/+	0-0/+	+	0	0/+	+	+	0/+	+	+	0/+	0	++	0

Abbreviations: M—matrix; P—perimatrix; E—epithelium; CT—connective tissue; HβD-2—human beta defensin 2; HβD-4—human beta defensin 4; IL-1—interleukin 1; IL-10—interleukin 10; Ki-67—proliferation marker; NF-κβ—nuclear factor kappa beta; VEGF—vascular endothelial growth factor; Shh—Sonic hedgehog gene protein; MMP-2—matrix metalloproteinase 2; MMP-9—matrix metalloproteinase 9; TIMP-2—tissue inhibitor of metalloproteinase-2; TIMP-4—tissue inhibitor of metalloproteinase-4; 0 = no positive structures, 0-0/+ = no to occasional positive structures, 0/+ = occasional positive structures, 0/+-+ = occasional-to-few positive structures, + = few positive structures, +/++ = few-to-moderate number of positive structures, ++ = moderate number of positive structures, ++/+++ = moderate-to-numerous positive structures, +++ = numerous positive cells, +++/++++ = numerous-to-abundant structures, ++++ = abundance of positive structures in the visual field.

Supplementary file

Table S2

Adult group correlations

			MMP-2 M	MMP-2 P	MMP-9M	MMP-9P	TIMP-2 M	TIMP-2 P	TIMP-4M	TIMP-4P	Shh M	Shh P	IL-1 M	IL-1 P	IL-10 M	IL-10 P	NFKB M	NFKB P	Ki-67 M	Ki-67 P	VEGF M	VEGF P	HBD-2 M	HBD-2 P	HBD-4 M	HBD-4 P
Spearman's rho	MMP-2 M	Correlation Coefficient	1.000	.574**	-0.199	-0.190	.484*	-0.057	0.183	-0.168	.719**	0.376	-0.220	-0.195	-0.150	-0.231	.399*	-0.202	0.230	0.078	-0.041	0.071	0.046	0.001	0.374	0.275
		Sig. (2-tailed)		0.003	0.339	0.362	0.014	0.788	0.382	0.422	0.000	0.064	0.290	0.349	0.475	0.267	0.048	0.332	0.268	0.709	0.844	0.737	0.828	0.996	0.065	0.184
		N	25	25	25	25	25	25	25	25	25	25	25	25	25	25	25	25	25	25	25	25	25	25	25	25
	MMP-2 P	Correlation Coefficient	.574**	1.000	-0.293	0.156	0.198	0.035	0.029	-0.114	.453*	.460*	-0.100	0.088	-0.111	0.131	0.169	0.090	0.078	0.275	0.014	0.219	-0.029	0.344	0.054	0.189
		Sig. (2-tailed)	0.003		0.156	0.456	0.344	0.866	0.892	0.587	0.023	0.021	0.633	0.674	0.596	0.533	0.419	0.670	0.712	0.184	0.948	0.292	0.891	0.092	0.798	0.367
		N	25	25	25	25	25	25	25	25	25	25	25	25	25	25	25	25	25	25	25	25	25	25	25	25
	MMP-9M	Correlation Coefficient	-0.199	-0.293	1.000	0.296	0.178	0.305	0.179	0.375	-0.209	-0.158	.426*	0.167	.458*	0.257	0.171	0.263	-0.235	-0.065	0.246	0.355	0.255	-0.013	-0.169	-0.140
		Sig. (2-tailed)	0.339	0.156		0.150	0.394	0.138	0.393	0.064	0.317	0.451	0.034	0.424	0.021	0.214	0.413	0.204	0.258	0.758	0.236	0.082	0.219	0.949	0.419	0.505
		N	25	25	25	25	25	25	25	25	25	25	25	25	25	25	25	25	25	25	25	25	25	25	25	25
	MMP-9P	Correlation Coefficient	-0.190	0.156	0.296	1.000	0.144	0.323	0.227	.664**	-0.033	0.128	.435*	.608**	0.357	.601**	0.287	.790**	0.086	.677**	0.324	0.361	.464*	.576**	-0.145	0.106
		Sig. (2-tailed)	0.362	0.456	0.150		0.493	0.115	0.276	0.000	0.877	0.541	0.030	0.001	0.080	0.001	0.165	0.000	0.683	0.000	0.114	0.077	0.019	0.003	0.488	0.613
		N	25	25	25	25	25	25	25	25	25	25	25	25	25	25	25	25	25	25	25	25	25	25	25	25
	TIMP-2 M	Correlation Coefficient	.484*	0.198	0.178	0.144	1.000	.676**	0.171	0.209	.478*	-0.103	0.289	0.070	0.349	0.266	.473*	0.005	-0.087	0.195	0.185	.563**	.416*	0.054	0.356	0.116
		Sig. (2-tailed)	0.014	0.344	0.394	0.493		0.000	0.413	0.316	0.016	0.625	0.161	0.739	0.088	0.199	0.017	0.980	0.681	0.350	0.376	0.003	0.038	0.797	0.081	0.582
		N	25	25	25	25	25	25	25	25	25	25	25	25	25	25	25	25	25	25	25	25	25	25	25	25
	TIMP-2 P	Correlation Coefficient	-0.057	0.035	0.305	0.323	.676**	1.000	-0.077	0.348	0.139	-0.232	.505**	0.386	.630**	.613**	0.307	0.383	-0.206	0.101	0.324	.503*	.523**	0.257	0.166	0.131
		Sig. (2-tailed)	0.788	0.866	0.138	0.115	0.000		0.713	0.088	0.507	0.264	0.010	0.057	0.001	0.001	0.136	0.059	0.324	0.630	0.114	0.010	0.007	0.215	0.428	0.531
		N	25	25	25	25	25	25	25	25	25	25	25	25	25	25	25	25	25	25	25	25	25	25	25	25
	TIMP-4M	Correlation Coefficient	0.183	0.029	0.179	0.227	0.171	-0.077	1.000	.431*	.436*	0.239	0.058	0.268	-0.194	-0.007	.540**	0.133	0.195	0.320	0.020	0.369	-0.082	0.072	0.104	0.005
		Sig. (2-tailed)	0.382	0.892	0.393	0.276	0.413	0.713		0.031	0.029	0.250	0.781	0.195	0.353	0.975	0.005	0.527	0.351	0.119	0.924	0.070	0.699	0.732	0.620	0.982
		N	25	25	25	25	25	25	25	25	25	25	25	25	25	25	25	25	25	25	25	25	25	25	25	25
	TIMP-4P	Correlation Coefficient	-0.168	-0.114	0.375	.664**	0.209	0.348	.431*	1.000	0.059	0.328	.457*	.637**	.425*	.638**	.422*	.749**	.402*	.651**	0.395	0.382	.404*	.545**	0.037	0.066
		Sig. (2-tailed)	0.422	0.587	0.064	0.000	0.316	0.088	0.031		0.781	0.109	0.022	0.001	0.034	0.001	0.036	0.000	0.046	0.000	0.051	0.059	0.045	0.005	0.859	0.752
		N	25	25	25	25	25	25	25	25	25	25	25	25	25	25	25	25	25	25	25	25	25	25	25	25
	Shh M	Correlation Coefficient	.719**	.453*	-0.209	-0.033	.478*	0.139	.436*	0.059	1.000	0.325	-0.078	0.127	0.035	-0.021	.549**	0.085	0.121	0.232	0.052	0.342	0.175	0.237	0.299	0.395
		Sig. (2-tailed)	0.000	0.023	0.317	0.877	0.016	0.507	0.029	0.781		0.113	0.710	0.544	0.867	0.921	0.005	0.687	0.565	0.264	0.805	0.094	0.403	0.254	0.146	0.051
		N	25	25	25	25	25	25	25	25	25	25	25	25	25	25	25	25	25	25	25	25	25	25	25	25
	Shh P	Correlation Coefficient	0.376	.460*	-0.158	0.128	-0.103	-0.232	0.239	0.328	0.325	1.000	-0.124	0.194	-0.073	0.117	0.322	0.308	.646**	0.350	0.114	0.047	-0.056	.527**	0.188	0.323
		Sig. (2-tailed)	0.064	0.021	0.451	0.541	0.625	0.264	0.250	0.109	0.113		0.554	0.352	0.729	0.578	0.117	0.134	0.000	0.086	0.586	0.823	0.789	0.007	0.368	0.115
		N	25	25	25	25	25	25	25	25	25	25	25	25	25	25	25	25	25	25	25	25	25	25	25	25
	IL-1 M	Correlation Coefficient	-0.220	-0.100	.426*	.435*	0.289	.505**	0.058	.457*	-0.078	-0.124	1.000	.557**	.813**	.762**	0.345	.519**	-0.100	0.277	0.388	0.148	.827**	0.352	-0.094	0.099
		Sig. (2-tailed)	0.290	0.633	0.034	0.030	0.161	0.010	0.781	0.022	0.710	0.554		0.004	0.000	0.000	0.091	0.008	0.634	0.180	0.055	0.480	0.000	0.084	0.656	0.636
		N	25	25	25	25	25	25	25	25	25	25	25	25	25	25	25	25	25	25	25	25	25	25	25	25
	IL-1 P	Correlation Coefficient	-0.195	0.088	0.167	.608**	0.070	0.386	0.268	.637**	0.127	0.194	.557**	1.000	0.391	.640**	0.381	.692**	0.067	.583**	0.128	0.292	.499*	.466*	-0.154	0.138
		Sig. (2-tailed)	0.349	0.674	0.424	0.001	0.739	0.057	0.195	0.001	0.544	0.352	0.004		0.053	0.001	0.060	0.000	0.749	0.002	0.541	0.156	0.011	0.019	0.462	0.511
		N	25	25	25	25	25	25	25	25	25	25	25	25	25	25	25	25	25	25	25	25	25	25	25	25
	IL-10 M	Correlation Coefficient	-0.150	-0.111	.458*	0.357	0.349	.630**	-0.194	.425*	0.035	-0.073	.813**	0.391	1.000	.801**	0.351	.554**	-0.038	0.099	.611**	0.233	.841**	.462*	-0.128	0.150
		Sig. (2-tailed)	0.475	0.596	0.021	0.080	0.088	0.001	0.353	0.034	0.867	0.729	0.000	0.053		0.000	0.086	0.004	0.855	0.639	0.001	0.262	0.000	0.020	0.543	0.476
		N	25	25	25	25	25	25	25	25	25	25	25	25	25	25	25	25	25	25	25	25	25	25	25	25
	IL-10 P	Correlation Coefficient	-0.231	0.131	0.257	.601**	0.266	.613**	-0.007	.638**	-0.021	0.117	.762**	.640**	.801**	1.000	.396*	.751**	0.175	.417*	.687**	0.357	.686**	.687**	-0.161	0.151
		Sig. (2-tailed)	0.267	0.533	0.214	0.001	0.199	0.001	0.975	0.001	0.921	0.578	0.000	0.001	0.000		0.050	0.000	0.404	0.038	0.000	0.079	0.000	0.000	0.443	0.471
		N	25	25	25	25	25	25	25	25	25	25	25	25	25	25	25	25	25	25	25	25	25	25	25	25
	NFKB M	Correlation Coefficient	.399*	0.169	0.171	0.287	.473*	0.307	.540**	.422*	.549**	0.322	0.345	0.381	0.351	.396*	1.000	0.380	0.155	0.330	.414*	0.350	.418*	0.315	0.317	0.265
		Sig. (2-tailed)	0.048	0.419	0.413	0.165	0.017	0.136	0.005	0.036	0.005	0.117	0.091	0.060	0.086	0.050		0.061	0.458	0.107	0.039	0.086	0.038	0.125	0.123	0.201
		N	25	25	25	25	25	25	25	25	25	25	25	25	25	25	25	25	25	25	25	25	25	25	25	25
	NFKB P	Correlation Coefficient	-0.202	0.090	0.263	.790**	0.005	0.383	0.133	.749**	0.085	0.308	.519**	.692**	.554**	.751**	0.380	1.000	0.253	.571**	.474*	0.198	.526**	.748**	-0.073	0.285
		Sig. (2-tailed)	0.332	0.670	0.204	0.000	0.980	0.059	0.527	0.000	0.687	0.134	0.008	0.000	0.004	0.000	0.061		0.222	0.003	0.017	0.342	0.007	0.000	0.729	0.168
		N	25	25	25	25	25	25	25	25	25	25	25	25	25	25	25	25	25	25	25	25	25	25	25	25
	Ki-67 M	Correlation Coefficient	0.230	0.078	-0.235	0.086	-0.087	-0.206	0.195	.402*	0.121	.646**	-0.100	0.067	-0.038	0.175	0.155	0.253	1.000	0.320	0.196	-0.215	0.012	.498*	0.224	0.365
		Sig. (2-tailed)	0.268	0.712	0.258	0.683	0.681	0.324	0.351	0.046	0.565	0.000	0.634	0.749	0.855	0.404	0.458	0.222		0.119	0.349	0.303	0.955	0.011	0.282	0.073
		N	25	25	25	25	25	25	25	25	25	25	25	25	25	25	25	25	25	25	25	25	25	25	25	25
	Ki-67 P	Correlation Coefficient	0.078	0.275	-0.065	.677**	0.195	0.101	0.320	.651**	0.232	0.350	0.277	.583**	0.099	.417*	0.330	.571**	0.320	1.000	0.153	0.295	0.349	.592**	0.119	0.248
		Sig. (2-tailed)	0.709	0.184	0.758	0.000	0.350	0.630	0.119	0.000	0.264	0.086	0.180	0.002	0.639	0.038	0.107	0.003	0.119		0.465	0.152	0.087	0.002	0.570	0.233
		N	25	25	25	25	25	25	25	25	25	25	25	25	25	25	25	25	25	25	25	25	25	25	25	25
	VEGF M	Correlation Coefficient	-0.041	0.014	0.246	0.324	0.185	0.324	0.020	0.395	0.052	0.114	0.388	0.128	.611**	.687**	.414*	.474*	0.196	0.153	1.000	.429*	0.385	.413*	-0.230	-0.038
		Sig. (2-tailed)	0.844	0.948	0.236	0.114	0.376	0.114	0.924	0.051	0.805	0.586	0.055	0.541	0.001	0.000	0.039	0.017	0.349	0.465		0.032	0.057	0.040	0.269	0.855
		N	25	25	25	25	25	25	25	25	25	25	25	25	25	25	25	25	25	25	25	25	25	25	25	25
	VEGF P	Correlation Coefficient	0.071	0.219	0.355	0.361	.563**	.503*	0.369	0.382	0.342	0.047	0.148	0.292	0.233	0.357	0.350	0.198	-0.215	0.295	.429*	1.000	0.106	0.145	-0.115	-0.147
		Sig. (2-tailed)	0.737	0.292	0.082	0.077	0.003	0.010	0.070	0.059	0.094	0.823	0.480	0.156	0.262	0.079	0.086	0.342	0.303	0.152	0.032		0.614	0.489	0.583	0.483
		N	25	25	25	25	25	25	25	25	25	25	25	25	25	25	25	25	25	25	25	25	25	25	25	25
	HBD-2 M	Correlation Coefficient	0.046	-0.029	0.255	.464*	.416*	.523**	-0.082	.404*	0.175	-0.056	.827**	.499*	.841**	.686**	.418*	.526**	0.012	0.349	0.385	0.106	1.000	.477*	-0.001	0.319
		Sig. (2-tailed)	0.828	0.891	0.219	0.019	0.038	0.007	0.699	0.045	0.403	0.789	0.000	0.011	0.000	0.000	0.038	0.007	0.955	0.087	0.057	0.614		0.016	0.998	0.120
		N	25	25	25	25	25	25	25	25	25	25	25	25	25	25	25	25	25	25	25	25	25	25	25	25
	HBD-2 P	Correlation Coefficient	0.001	0.344	-0.013	.576**	0.054	0.257	0.072	.545**	0.237	.527**	0.352	.466*	.462*	.687**	0.315	.748**	.498*	.592**	.413*	0.145	.477*	1.000	0.029	.537**
		Sig. (2-tailed)	0.996	0.092	0.949	0.003	0.797	0.215	0.732	0.005	0.254	0.007	0.084	0.019	0.020	0.000	0.125	0.000	0.011	0.002	0.040	0.489	0.016		0.889	0.006
		N	25	25	25	25	25	25	25	25	25	25	25	25	25	25	25	25	25	25	25	25	25	25	25	25
	HBD-4 M	Correlation Coefficient	0.374	0.054	-0.169	-0.145	0.356	0.166	0.104	0.037	0.299	0.188	-0.094	-0.154	-0.128	-0.161	0.317	-0.073	0.224	0.119	-0.230	-0.115	-0.001	0.029	1.000	.655**
		Sig. (2-tailed)	0.065	0.798	0.419	0.488	0.081	0.428	0.620	0.859	0.146	0.368	0.656	0.462	0.543	0.443	0.123	0.729	0.282	0.570	0.269	0.583	0.998	0.889		0.000
		N	25	25	25	25	25	25	25	25	25	25	25	25	25	25	25	25	25	25	25	25	25	25	25	25
	HBD-4 P	Correlation Coefficient	0.275	0.189	-0.140	0.106	0.116	0.131	0.005	0.066	0.395	0.323	0.099	0.138	0.150	0.151	0.265	0.285	0.365	0.248	-0.038	-0.147	0.319	.537**	.655**	1.000
		Sig. (2-tailed)	0.184	0.367	0.505	0.613	0.582	0.531	0.982	0.752	0.051	0.115	0.636	0.511	0.476	0.471	0.201	0.168	0.073	0.233	0.855	0.483	0.120	0.006	0.000	
		N	25	25	25	25	25	25	25	25	25	25	25	25	25	25	25	25	25	25	25	25	25	25	25	25

**. Correlation is significant at the 0.01 level (2-tailed).

*. Correlation is significant at the 0.05 level (2-tailed).

Table S3

Children group correlations

			MMP-2 M	MMP-2 P	MMP-9M	MMP-9P	TIMP-2 M	TIMP-2 P	TIMP-4M	TIMP-4P	Shh M	Shh P	IL-1 M	IL-1 P	IL-10 M	IL-10 P	NFKB M	NFKB P	Ki-67 M	Ki-67 P	VEGF M	VEGF P	HBD-2 M	HBD-2 P	HBD-4 M	HBD-4 P
Spearman's rho	MMP-2 M	Correlation Coefficient	1.000	.803**	0.312	.415*	.622**	.535**	.550**	0.368	.786**	.439*	.499*	0.236	.420*	0.100	.677**	.510**	.516**	0.278	0.188	0.238	.475*	0.332	0.334	0.066
		Sig. (2-tailed)		0.000	0.129	0.039	0.001	0.006	0.004	0.071	0.000	0.028	0.011	0.256	0.037	0.633	0.000	0.009	0.008	0.178	0.367	0.253	0.016	0.105	0.103	0.753
		N	25	25	25	25	25	25	25	25	25	25	25	25	25	25	25	25	25	25	25	25	25	25	25	25
	MMP-2 P	Correlation Coefficient	.803**	1.000	0.227	.451*	.459*	.509**	.589**	0.386	.786**	.653**	0.342	0.276	0.327	0.189	.629**	.643**	.545**	0.354	0.284	0.364	0.376	.539**	0.159	0.109
		Sig. (2-tailed)	0.000		0.276	0.024	0.021	0.009	0.002	0.057	0.000	0.000	0.094	0.182	0.110	0.365	0.001	0.001	0.005	0.082	0.169	0.073	0.064	0.005	0.448	0.604
		N	25	25	25	25	25	25	25	25	25	25	25	25	25	25	25	25	25	25	25	25	25	25	25	25
	MMP-9M	Correlation Coefficient	0.312	0.227	1.000	.710**	.436*	0.346	.481*	.528**	.473*	0.316	.549**	.604**	.418*	0.332	.522**	.531**	.560**	0.373	.561**	.525**	.562**	0.225	0.127	0.027
		Sig. (2-tailed)	0.129	0.276		0.000	0.029	0.090	0.015	0.007	0.017	0.124	0.004	0.001	0.038	0.105	0.007	0.006	0.004	0.067	0.004	0.007	0.003	0.280	0.545	0.898
		N	25	25	25	25	25	25	25	25	25	25	25	25	25	25	25	25	25	25	25	25	25	25	25	25
	MMP-9P	Correlation Coefficient	.415*	.451*	.710**	1.000	.463*	.549**	.585**	.490*	.558**	.461*	.642**	.714**	.397*	.468*	.630**	.614**	.623**	.624**	.550**	.571**	.487*	0.286	0.199	0.173
		Sig. (2-tailed)	0.039	0.024	0.000		0.020	0.005	0.002	0.013	0.004	0.020	0.001	0.000	0.050	0.018	0.001	0.001	0.001	0.001	0.004	0.003	0.014	0.165	0.340	0.409
		N	25	25	25	25	25	25	25	25	25	25	25	25	25	25	25	25	25	25	25	25	25	25	25	25
	TIMP-2 M	Correlation Coefficient	.622**	.459*	.436*	.463*	1.000	.685**	0.220	0.186	.537**	0.287	.597**	.407*	.488*	0.206	.504*	.462*	.420*	0.118	0.302	0.294	.505*	0.277	.409*	-0.097
		Sig. (2-tailed)	0.001	0.021	0.029	0.020		0.000	0.292	0.374	0.006	0.164	0.002	0.044	0.013	0.323	0.010	0.020	0.037	0.573	0.142	0.154	0.010	0.179	0.042	0.645
		N	25	25	25	25	25	25	25	25	25	25	25	25	25	25	25	25	25	25	25	25	25	25	25	25
	TIMP-2 P	Correlation Coefficient	.535**	.509**	0.346	.549**	.685**	1.000	0.355	0.303	.613**	.442*	.457*	.474*	.423*	0.370	.560**	.548**	.413*	0.237	.453*	.445*	.526**	0.380	0.392	0.177
		Sig. (2-tailed)	0.006	0.009	0.090	0.005	0.000		0.081	0.140	0.001	0.027	0.022	0.017	0.035	0.069	0.004	0.005	0.040	0.253	0.023	0.026	0.007	0.061	0.052	0.398
		N	25	25	25	25	25	25	25	25	25	25	25	25	25	25	25	25	25	25	25	25	25	25	25	25
	TIMP-4M	Correlation Coefficient	.550**	.589**	.481*	.585**	0.220	0.355	1.000	.841**	.681**	.566**	.634**	.575**	.522**	.605**	.738**	.485*	.569**	.590**	.492*	.690**	.608**	.500*	-0.104	0.069
		Sig. (2-tailed)	0.004	0.002	0.015	0.002	0.292	0.081		0.000	0.000	0.003	0.001	0.003	0.008	0.001	0.000	0.014	0.003	0.002	0.012	0.000	0.001	0.011	0.619	0.745
		N	25	25	25	25	25	25	25	25	25	25	25	25	25	25	25	25	25	25	25	25	25	25	25	25
	TIMP-4P	Correlation Coefficient	0.368	0.386	.528**	.490*	0.186	0.303	.841**	1.000	.475*	0.367	.641**	.663**	.638**	.721**	.654**	.457*	.481*	.414*	.628**	.678**	.568**	.397*	-0.185	0.018
		Sig. (2-tailed)	0.071	0.057	0.007	0.013	0.374	0.140	0.000		0.016	0.071	0.001	0.000	0.001	0.000	0.000	0.022	0.015	0.040	0.001	0.000	0.003	0.049	0.376	0.930
		N	25	25	25	25	25	25	25	25	25	25	25	25	25	25	25	25	25	25	25	25	25	25	25	25
	Shh M	Correlation Coefficient	.786**	.786**	.473*	.558**	.537**	.613**	.681**	.475*	1.000	.813**	.472*	0.393	.444*	0.232	.753**	.702**	.803**	.575**	0.278	0.384	.539**	.515**	.437*	0.278
		Sig. (2-tailed)	0.000	0.000	0.017	0.004	0.006	0.001	0.000	0.016		0.000	0.017	0.052	0.026	0.264	0.000	0.000	0.000	0.003	0.179	0.058	0.005	0.008	0.029	0.178
		N	25	25	25	25	25	25	25	25	25	25	25	25	25	25	25	25	25	25	25	25	25	25	25	25
	Shh P	Correlation Coefficient	.439*	.653**	0.316	.461*	0.287	.442*	.566**	0.367	.813**	1.000	0.320	0.271	0.197	0.273	.570**	.530**	.746**	.667**	0.152	0.278	0.283	.428*	0.367	0.284
		Sig. (2-tailed)	0.028	0.000	0.124	0.020	0.164	0.027	0.003	0.071	0.000		0.119	0.191	0.344	0.187	0.003	0.006	0.000	0.000	0.467	0.178	0.171	0.033	0.071	0.169
		N	25	25	25	25	25	25	25	25	25	25	25	25	25	25	25	25	25	25	25	25	25	25	25	25
	IL-1 M	Correlation Coefficient	.499*	0.342	.549**	.642**	.597**	.457*	.634**	.641**	.472*	0.320	1.000	.716**	.709**	.720**	.827**	.406*	.597**	.493*	.510**	.574**	.700**	0.377	0.207	0.101
		Sig. (2-tailed)	0.011	0.094	0.004	0.001	0.002	0.022	0.001	0.001	0.017	0.119		0.000	0.000	0.000	0.000	0.044	0.002	0.012	0.009	0.003	0.000	0.063	0.320	0.632
		N	25	25	25	25	25	25	25	25	25	25	25	25	25	25	25	25	25	25	25	25	25	25	25	25
	IL-1 P	Correlation Coefficient	0.236	0.276	.604**	.714**	.407*	.474*	.575**	.663**	0.393	0.271	.716**	1.000	.620**	.694**	.647**	.510**	.442*	.441*	.664**	.714**	.630**	0.369	0.075	0.212
		Sig. (2-tailed)	0.256	0.182	0.001	0.000	0.044	0.017	0.003	0.000	0.052	0.191	0.000		0.001	0.000	0.000	0.009	0.027	0.027	0.000	0.000	0.001	0.069	0.722	0.310
		N	25	25	25	25	25	25	25	25	25	25	25	25	25	25	25	25	25	25	25	25	25	25	25	25
	IL-10 M	Correlation Coefficient	.420*	0.327	.418*	.397*	.488*	.423*	.522**	.638**	.444*	0.197	.709**	.620**	1.000	.668**	.677**	.536**	.558**	.437*	.559**	.422*	.828**	.677**	0.082	0.140
		Sig. (2-tailed)	0.037	0.110	0.038	0.050	0.013	0.035	0.008	0.001	0.026	0.344	0.000	0.001		0.000	0.000	0.006	0.004	0.029	0.004	0.036	0.000	0.000	0.698	0.506
		N	25	25	25	25	25	25	25	25	25	25	25	25	25	25	25	25	25	25	25	25	25	25	25	25
	IL-10 P	Correlation Coefficient	0.100	0.189	0.332	.468*	0.206	0.370	.605**	.721**	0.232	0.273	.720**	.694**	.668**	1.000	.602**	0.316	0.376	0.372	.696**	.674**	.592**	.516**	-0.163	0.198
		Sig. (2-tailed)	0.633	0.365	0.105	0.018	0.323	0.069	0.001	0.000	0.264	0.187	0.000	0.000	0.000		0.001	0.124	0.064	0.067	0.000	0.000	0.002	0.008	0.438	0.343
		N	25	25	25	25	25	25	25	25	25	25	25	25	25	25	25	25	25	25	25	25	25	25	25	25
	NFKB M	Correlation Coefficient	.677**	.629**	.522**	.630**	.504*	.560**	.738**	.654**	.753**	.570**	.827**	.647**	.677**	.602**	1.000	.680**	.726**	.588**	.595**	.614**	.750**	.570**	0.188	0.305
		Sig. (2-tailed)	0.000	0.001	0.007	0.001	0.010	0.004	0.000	0.000	0.000	0.003	0.000	0.000	0.000	0.001		0.000	0.000	0.002	0.002	0.001	0.000	0.003	0.368	0.138
		N	25	25	25	25	25	25	25	25	25	25	25	25	25	25	25	25	25	25	25	25	25	25	25	25
	NFKB P	Correlation Coefficient	.510**	.643**	.531**	.614**	.462*	.548**	.485*	.457*	.702**	.530**	.406*	.510**	.536**	0.316	.680**	1.000	.678**	.494*	.637**	.473*	.621**	.635**	0.154	0.271
		Sig. (2-tailed)	0.009	0.001	0.006	0.001	0.020	0.005	0.014	0.022	0.000	0.006	0.044	0.009	0.006	0.124	0.000		0.000	0.012	0.001	0.017	0.001	0.001	0.461	0.190
		N	25	25	25	25	25	25	25	25	25	25	25	25	25	25	25	25	25	25	25	25	25	25	25	25
	Ki-67 M	Correlation Coefficient	.516**	.545**	.560**	.623**	.420*	.413*	.569**	.481*	.803**	.746**	.597**	.442*	.558**	0.376	.726**	.678**	1.000	.780**	0.311	0.293	.595**	.520**	.426*	.426*
		Sig. (2-tailed)	0.008	0.005	0.004	0.001	0.037	0.040	0.003	0.015	0.000	0.000	0.002	0.027	0.004	0.064	0.000	0.000		0.000	0.130	0.155	0.002	0.008	0.034	0.034
		N	25	25	25	25	25	25	25	25	25	25	25	25	25	25	25	25	25	25	25	25	25	25	25	25
	Ki-67 P	Correlation Coefficient	0.278	0.354	0.373	.624**	0.118	0.237	.590**	.414*	.575**	.667**	.493*	.441*	.437*	0.372	.588**	.494*	.780**	1.000	0.235	0.257	.461*	0.366	0.323	0.386
		Sig. (2-tailed)	0.178	0.082	0.067	0.001	0.573	0.253	0.002	0.040	0.003	0.000	0.012	0.027	0.029	0.067	0.002	0.012	0.000		0.258	0.216	0.020	0.072	0.115	0.057
		N	25	25	25	25	25	25	25	25	25	25	25	25	25	25	25	25	25	25	25	25	25	25	25	25
	VEGF M	Correlation Coefficient	0.188	0.284	.561**	.550**	0.302	.453*	.492*	.628**	0.278	0.152	.510**	.664**	.559**	.696**	.595**	.637**	0.311	0.235	1.000	.745**	.639**	.509**	-0.343	0.044
		Sig. (2-tailed)	0.367	0.169	0.004	0.004	0.142	0.023	0.012	0.001	0.179	0.467	0.009	0.000	0.004	0.000	0.002	0.001	0.130	0.258		0.000	0.001	0.009	0.093	0.835
		N	25	25	25	25	25	25	25	25	25	25	25	25	25	25	25	25	25	25	25	25	25	25	25	25
	VEGF P	Correlation Coefficient	0.238	0.364	.525**	.571**	0.294	.445*	.690**	.678**	0.384	0.278	.574**	.714**	.422*	.674**	.614**	.473*	0.293	0.257	.745**	1.000	.595**	0.390	-0.176	0.144
		Sig. (2-tailed)	0.253	0.073	0.007	0.003	0.154	0.026	0.000	0.000	0.058	0.178	0.003	0.000	0.036	0.000	0.001	0.017	0.155	0.216	0.000		0.002	0.054	0.400	0.492
		N	25	25	25	25	25	25	25	25	25	25	25	25	25	25	25	25	25	25	25	25	25	25	25	25
	HBD-2 M	Correlation Coefficient	.475*	0.376	.562**	.487*	.505*	.526**	.608**	.568**	.539**	0.283	.700**	.630**	.828**	.592**	.750**	.621**	.595**	.461*	.639**	.595**	1.000	.748**	0.081	0.281
		Sig. (2-tailed)	0.016	0.064	0.003	0.014	0.010	0.007	0.001	0.003	0.005	0.171	0.000	0.001	0.000	0.002	0.000	0.001	0.002	0.020	0.001	0.002		0.000	0.701	0.174
		N	25	25	25	25	25	25	25	25	25	25	25	25	25	25	25	25	25	25	25	25	25	25	25	25
	HBD-2 P	Correlation Coefficient	0.332	.539**	0.225	0.286	0.277	0.380	.500*	.397*	.515**	.428*	0.377	0.369	.677**	.516**	.570**	.635**	.520**	0.366	.509**	0.390	.748**	1.000	-0.102	0.201
		Sig. (2-tailed)	0.105	0.005	0.280	0.165	0.179	0.061	0.011	0.049	0.008	0.033	0.063	0.069	0.000	0.008	0.003	0.001	0.008	0.072	0.009	0.054	0.000		0.627	0.336
		N	25	25	25	25	25	25	25	25	25	25	25	25	25	25	25	25	25	25	25	25	25	25	25	25
	HBD-4 M	Correlation Coefficient	0.334	0.159	0.127	0.199	.409*	0.392	-0.104	-0.185	.437*	0.367	0.207	0.075	0.082	-0.163	0.188	0.154	.426*	0.323	-0.343	-0.176	0.081	-0.102	1.000	0.380
		Sig. (2-tailed)	0.103	0.448	0.545	0.340	0.042	0.052	0.619	0.376	0.029	0.071	0.320	0.722	0.698	0.438	0.368	0.461	0.034	0.115	0.093	0.400	0.701	0.627		0.061
		N	25	25	25	25	25	25	25	25	25	25	25	25	25	25	25	25	25	25	25	25	25	25	25	25
	HBD-4 P	Correlation Coefficient	0.066	0.109	0.027	0.173	-0.097	0.177	0.069	0.018	0.278	0.284	0.101	0.212	0.140	0.198	0.305	0.271	.426*	0.386	0.044	0.144	0.281	0.201	0.380	1.000
		Sig. (2-tailed)	0.753	0.604	0.898	0.409	0.645	0.398	0.745	0.930	0.178	0.169	0.632	0.310	0.506	0.343	0.138	0.190	0.034	0.057	0.835	0.492	0.174	0.336	0.061	
		N	25	25	25	25	25	25	25	25	25	25	25	25	25	25	25	25	25	25	25	25	25	25	25	25

**. Correlation is significant at the 0.01 level (2-tailed).

*. Correlation is significant at the 0.05 level (2-tailed).

Table S4

Control group correlations

			MMP-2 M	MMP-2 P	MMP-9M	MMP-9P	TIMP-2 M	TIMP-2 P	TIMP-4M	TIMP-4P	Shh M	Shh P	IL-1 M	IL-1 P	IL-10 M	IL-10 P	NFKB M	NFKB P	Ki-67 M	Ki-67 P	VEGF M	VEGF P	HBD-2 M	HBD-2 P	HBD-4 M	HBD-4 P
Spearman's rho	MMP-2 M	Correlation Coefficient	1.000	0.520	0.472	-0.271	.833*	0.396	0.547	0.416	0.593	0.385	0.009	-.808*	0.346	-0.061	.785*	0.591	0.644	0.644	0.577	0.448	0.664	0.668	0.023	-0.010
		Sig. (2-tailed)		0.232	0.285	0.557	0.020	0.380	0.204	0.353	0.161	0.393	0.984	0.028	0.447	0.897	0.036	0.163	0.118	0.118	0.175	0.314	0.104	0.101	0.962	0.983
		N	7	7	7	7	7	7	7	7	7	7	7	7	7	7	7	7	7	7	7	7	7	7	7	7
	MMP-2 P	Correlation Coefficient	0.520	1.000	0.540	0.113	0.312	0.683	0.424	0.228	0.312	0.569	0.105	-0.471	-0.105	-0.342	0.525	0.535	0.258	0.258	0.432	0.321	0.629	0.441	-0.509	-0.569
		Sig. (2-tailed)	0.232		0.211	0.810	0.496	0.091	0.344	0.623	0.496	0.182	0.823	0.286	0.823	0.453	0.227	0.216	0.576	0.576	0.333	0.483	0.130	0.322	0.243	0.182
		N	7	7	7	7	7	7	7	7	7	7	7	7	7	7	7	7	7	7	7	7	7	7	7	7
	MMP-9M	Correlation Coefficient	0.472	0.540	1.000	0.292	0.683	0.601	0.726	.769*	.924**	.896**	-0.612	-0.153	0.690	0.474	.777*	.772*	0.251	0.251	0.670	0.535	0.728	0.204	0.024	-0.011
		Sig. (2-tailed)	0.285	0.211		0.525	0.091	0.154	0.065	0.043	0.003	0.006	0.144	0.744	0.086	0.282	0.040	0.042	0.587	0.587	0.100	0.216	0.063	0.661	0.960	0.982
		N	7	7	7	7	7	7	7	7	7	7	7	7	7	7	7	7	7	7	7	7	7	7	7	7
	MMP-9P	Correlation Coefficient	-0.271	0.113	0.292	1.000	-0.050	0.231	0.429	0.231	0.201	0.231	-0.557	0.159	0.152	0.231	0.223	0.434	-0.611	-0.611	0.156	-0.062	0.152	-0.128	0.516	0.385
		Sig. (2-tailed)	0.557	0.810	0.525		0.915	0.619	0.336	0.619	0.666	0.619	0.194	0.733	0.745	0.619	0.631	0.331	0.145	0.145	0.738	0.895	0.745	0.785	0.236	0.394
		N	7	7	7	7	7	7	7	7	7	7	7	7	7	7	7	7	7	7	7	7	7	7	7	7
	TIMP-2 M	Correlation Coefficient	.833*	0.312	0.683	-0.050	1.000	0.152	0.585	0.639	.824*	0.629	-0.224	-0.441	0.598	0.183	.925**	0.619	0.564	0.564	0.433	0.238	0.495	0.314	0.249	0.122
		Sig. (2-tailed)	0.020	0.496	0.091	0.915		0.745	0.168	0.122	0.023	0.130	0.629	0.322	0.156	0.695	0.003	0.138	0.188	0.188	0.332	0.607	0.258	0.492	0.590	0.795
		N	7	7	7	7	7	7	7	7	7	7	7	7	7	7	7	7	7	7	7	7	7	7	7	7
	TIMP-2 P	Correlation Coefficient	0.396	0.683	0.601	0.231	0.152	1.000	0.620	0.333	0.456	0.433	-0.461	-0.483	0.338	0.300	0.338	0.658	0.000	0.000	.885**	.845*	.921**	0.645	-0.149	-0.033
		Sig. (2-tailed)	0.380	0.091	0.154	0.619	0.745		0.137	0.465	0.303	0.331	0.298	0.272	0.459	0.513	0.459	0.108	1.000	1.000	0.008	0.017	0.003	0.117	0.750	0.943
		N	7	7	7	7	7	7	7	7	7	7	7	7	7	7	7	7	7	7	7	7	7	7	7	7
	TIMP-4M	Correlation Coefficient	0.547	0.424	0.726	0.429	0.585	0.620	1.000	.868*	.811*	0.393	-0.705	-0.599	0.476	0.310	.781*	.990**	0.328	0.328	0.706	0.641	0.724	0.240	0.277	0.434
		Sig. (2-tailed)	0.204	0.344	0.065	0.336	0.168	0.137		0.011	0.027	0.383	0.077	0.155	0.280	0.499	0.038	0.000	0.472	0.472	0.076	0.121	0.066	0.604	0.547	0.330
		N	7	7	7	7	7	7	7	7	7	7	7	7	7	7	7	7	7	7	7	7	7	7	7	7
	TIMP-4P	Correlation Coefficient	0.416	0.228	.769*	0.231	0.639	0.333	.868*	1.000	.882**	0.456	-0.686	-0.322	0.522	0.367	0.747	.845*	0.529	0.529	0.464	0.491	0.471	-0.129	0.149	0.344
		Sig. (2-tailed)	0.353	0.623	0.043	0.619	0.122	0.465	0.011		0.009	0.304	0.089	0.481	0.229	0.419	0.053	0.017	0.222	0.222	0.295	0.264	0.286	0.783	0.750	0.449
		N	7	7	7	7	7	7	7	7	7	7	7	7	7	7	7	7	7	7	7	7	7	7	7	7
	Shh M	Correlation Coefficient	0.593	0.312	.924**	0.201	.824*	0.456	.811*	.882**	1.000	0.730	-0.682	-0.294	.794*	0.548	.851*	.819*	0.403	0.403	0.674	0.562	0.682	0.196	0.249	0.274
		Sig. (2-tailed)	0.161	0.496	0.003	0.666	0.023	0.303	0.027	0.009		0.062	0.091	0.522	0.033	0.203	0.015	0.024	0.371	0.371	0.097	0.189	0.091	0.673	0.590	0.552
		N	7	7	7	7	7	7	7	7	7	7	7	7	7	7	7	7	7	7	7	7	7	7	7	7
	Shh P	Correlation Coefficient	0.385	0.569	.896**	0.231	0.629	0.433	0.393	0.456	0.730	1.000	-0.317	0.081	0.584	0.333	0.665	0.480	0.088	0.088	0.464	0.240	0.563	0.215	-0.075	-0.300
		Sig. (2-tailed)	0.393	0.182	0.006	0.619	0.130	0.331	0.383	0.304	0.062		0.488	0.864	0.169	0.465	0.103	0.276	0.851	0.851	0.295	0.604	0.188	0.643	0.874	0.513
		N	7	7	7	7	7	7	7	7	7	7	7	7	7	7	7	7	7	7	7	7	7	7	7	7
	IL-1 M	Correlation Coefficient	0.009	0.105	-0.612	-0.557	-0.224	-0.461	-0.705	-0.686	-0.682	-0.317	1.000	0.000	-0.736	-.829*	-0.330	-0.644	0.163	0.163	-0.622	-0.596	-0.500	0.000	-0.527	-0.676
		Sig. (2-tailed)	0.984	0.823	0.144	0.194	0.629	0.298	0.077	0.089	0.091	0.488		1.000	0.059	0.021	0.470	0.118	0.728	0.728	0.136	0.158	0.253	1.000	0.225	0.096
		N	7	7	7	7	7	7	7	7	7	7	7	7	7	7	7	7	7	7	7	7	7	7	7	7
	IL-1 P	Correlation Coefficient	-.808*	-0.471	-0.153	0.159	-0.441	-0.483	-0.599	-0.322	-0.294	0.081	0.000	1.000	0.000	0.242	-0.519	-0.605	-0.548	-0.548	-0.535	-0.529	-0.593	-0.624	0.000	-0.161
		Sig. (2-tailed)	0.028	0.286	0.744	0.733	0.322	0.272	0.155	0.481	0.522	0.864	1.000		1.000	0.602	0.232	0.150	0.203	0.203	0.216	0.222	0.160	0.135	1.000	0.730
		N	7	7	7	7	7	7	7	7	7	7	7	7	7	7	7	7	7	7	7	7	7	7	7	7
	IL-10 M	Correlation Coefficient	0.346	-0.105	0.690	0.152	0.598	0.338	0.476	0.522	.794*	0.584	-0.736	0.000	1.000	.891**	0.481	0.452	0.000	0.000	0.680	0.539	0.575	0.317	0.527	0.471
		Sig. (2-tailed)	0.447	0.823	0.086	0.745	0.156	0.459	0.280	0.229	0.033	0.169	0.059	1.000		0.007	0.274	0.309	1.000	1.000	0.093	0.212	0.176	0.488	0.225	0.286
		N	7	7	7	7	7	7	7	7	7	7	7	7	7	7	7	7	7	7	7	7	7	7	7	7
	IL-10 P	Correlation Coefficient	-0.061	-0.342	0.474	0.231	0.183	0.300	0.310	0.367	0.548	0.333	-.829*	0.242	.891**	1.000	0.092	0.250	-0.265	-0.265	0.569	0.532	0.399	0.129	0.522	0.567
		Sig. (2-tailed)	0.897	0.453	0.282	0.619	0.695	0.513	0.499	0.419	0.203	0.465	0.021	0.602	0.007		0.844	0.588	0.566	0.566	0.182	0.219	0.375	0.783	0.230	0.185
		N	7	7	7	7	7	7	7	7	7	7	7	7	7	7	7	7	7	7	7	7	7	7	7	7
	NFKB M	Correlation Coefficient	.785*	0.525	.777*	0.223	.925**	0.338	.781*	0.747	.851*	0.665	-0.330	-0.519	0.481	0.092	1.000	.827*	0.488	0.488	0.495	0.289	0.594	0.278	0.206	0.113
		Sig. (2-tailed)	0.036	0.227	0.040	0.631	0.003	0.459	0.038	0.053	0.015	0.103	0.470	0.232	0.274	0.844		0.022	0.267	0.267	0.258	0.530	0.159	0.547	0.658	0.810
		N	7	7	7	7	7	7	7	7	7	7	7	7	7	7	7	7	7	7	7	7	7	7	7	7
	NFKB P	Correlation Coefficient	0.591	0.535	.772*	0.434	0.619	0.658	.990**	.845*	.819*	0.480	-0.644	-0.605	0.452	0.250	.827*	1.000	0.331	0.331	0.713	0.618	.760*	0.283	0.210	0.324
		Sig. (2-tailed)	0.163	0.216	0.042	0.331	0.138	0.108	0.000	0.017	0.024	0.276	0.118	0.150	0.309	0.588	0.022		0.468	0.468	0.072	0.139	0.048	0.539	0.651	0.479
		N	7	7	7	7	7	7	7	7	7	7	7	7	7	7	7	7	7	7	7	7	7	7	7	7
	Ki-67 M	Correlation Coefficient	0.644	0.258	0.251	-0.611	0.564	0.000	0.328	0.529	0.403	0.088	0.163	-0.548	0.000	-0.265	0.488	0.331	1.000	1.000**	0.084	0.249	0.163	0.000	-0.394	-0.176
		Sig. (2-tailed)	0.118	0.576	0.587	0.145	0.188	1.000	0.472	0.222	0.371	0.851	0.728	0.203	1.000	0.566	0.267	0.468			0.858	0.591	0.728	1.000	0.381	0.705
		N	7	7	7	7	7	7	7	7	7	7	7	7	7	7	7	7	7	7	7	7	7	7	7	7
	Ki-67 P	Correlation Coefficient	0.644	0.258	0.251	-0.611	0.564	0.000	0.328	0.529	0.403	0.088	0.163	-0.548	0.000	-0.265	0.488	0.331	1.000**	1.000	0.084	0.249	0.163	0.000	-0.394	-0.176
		Sig. (2-tailed)	0.118	0.576	0.587	0.145	0.188	1.000	0.472	0.222	0.371	0.851	0.728	0.203	1.000	0.566	0.267	0.468			0.858	0.591	0.728	1.000	0.381	0.705
		N	7	7	7	7	7	7	7	7	7	7	7	7	7	7	7	7	7	7	7	7	7	7	7	7
	VEGF M	Correlation Coefficient	0.577	0.432	0.670	0.156	0.433	.885**	0.706	0.464	0.674	0.464	-0.622	-0.535	0.680	0.569	0.495	0.713	0.084	0.084	1.000	.911**	.971**	0.735	0.189	0.274
		Sig. (2-tailed)	0.175	0.333	0.100	0.738	0.332	0.008	0.076	0.295	0.097	0.295	0.136	0.216	0.093	0.182	0.258	0.072	0.858	0.858		0.004	0.000	0.060	0.686	0.552
		N	7	7	7	7	7	7	7	7	7	7	7	7	7	7	7	7	7	7	7	7	7	7	7	7
	VEGF P	Correlation Coefficient	0.448	0.321	0.535	-0.062	0.238	.845*	0.641	0.491	0.562	0.240	-0.596	-0.529	0.539	0.532	0.289	0.618	0.249	0.249	.911**	1.000	.856*	0.566	-0.023	0.250
		Sig. (2-tailed)	0.314	0.483	0.216	0.895	0.607	0.017	0.121	0.264	0.189	0.604	0.158	0.222	0.212	0.219	0.530	0.139	0.591	0.591	0.004		0.014	0.185	0.960	0.588
		N	7	7	7	7	7	7	7	7	7	7	7	7	7	7	7	7	7	7	7	7	7	7	7	7
	HBD-2 M	Correlation Coefficient	0.664	0.629	0.728	0.152	0.495	.921**	0.724	0.471	0.682	0.563	-0.500	-0.593	0.575	0.399	0.594	.760*	0.163	0.163	.971**	.856*	1.000	0.753	0.046	0.092
		Sig. (2-tailed)	0.104	0.130	0.063	0.745	0.258	0.003	0.066	0.286	0.091	0.188	0.253	0.160	0.176	0.375	0.159	0.048	0.728	0.728	0.000	0.014		0.051	0.922	0.844
		N	7	7	7	7	7	7	7	7	7	7	7	7	7	7	7	7	7	7	7	7	7	7	7	7
	HBD-2 P	Correlation Coefficient	0.668	0.441	0.204	-0.128	0.314	0.645	0.240	-0.129	0.196	0.215	0.000	-0.624	0.317	0.129	0.278	0.283	0.000	0.000	0.735	0.566	0.753	1.000	0.096	0.000
		Sig. (2-tailed)	0.101	0.322	0.661	0.785	0.492	0.117	0.604	0.783	0.673	0.643	1.000	0.135	0.488	0.783	0.547	0.539	1.000	1.000	0.060	0.185	0.051		0.837	1.000
		N	7	7	7	7	7	7	7	7	7	7	7	7	7	7	7	7	7	7	7	7	7	7	7	7
	HBD-4 M	Correlation Coefficient	0.023	-0.509	0.024	0.516	0.249	-0.149	0.277	0.149	0.249	-0.075	-0.527	0.000	0.527	0.522	0.206	0.210	-0.394	-0.394	0.189	-0.023	0.046	0.096	1.000	.870*
		Sig. (2-tailed)	0.962	0.243	0.960	0.236	0.590	0.750	0.547	0.750	0.590	0.874	0.225	1.000	0.225	0.230	0.658	0.651	0.381	0.381	0.686	0.960	0.922	0.837		0.011
		N	7	7	7	7	7	7	7	7	7	7	7	7	7	7	7	7	7	7	7	7	7	7	7	7
	HBD-4 P	Correlation Coefficient	-0.010	-0.569	-0.011	0.385	0.122	-0.033	0.434	0.344	0.274	-0.300	-0.676	-0.161	0.471	0.567	0.113	0.324	-0.176	-0.176	0.274	0.250	0.092	0.000	.870*	1.000
		Sig. (2-tailed)	0.983	0.182	0.982	0.394	0.795	0.943	0.330	0.449	0.552	0.513	0.096	0.730	0.286	0.185	0.810	0.479	0.705	0.705	0.552	0.588	0.844	1.000	0.011	
		N	7	7	7	7	7	7	7	7	7	7	7	7	7	7	7	7	7	7	7	7	7	7	7	7

*. Correlation is significant at the 0.05 level (2-tailed).

**. Correlation is significant at the 0.01 level (2-tailed).
